# Conserved Kir channel mechanisms governing intrinsic excitability in human and rodent parvalbumin neurons

**DOI:** 10.1038/s42003-026-10063-9

**Published:** 2026-04-13

**Authors:** Szabina Furdan, Abdennour Douida, Emoke Bakos, Ádám Tiszlavicz, Kolos Nemes, Lőrinc Sándor Pongor, Krisztián Péli, Gabor Molnar, Gabor Tamas, Daphne Welter, Jonathan Landry, Bálint H. Kovács, Miklós Erdélyi, Balazs Bende, Gabor Hutoczki, Attila Papp, Pal Barzo, Vladimir Benes, Attila Szucs, Viktor Szegedi, Karri Lamsa

**Affiliations:** 1 Hungarian Center of Excellence for Molecular Medicine Research Group for Human neuron physiology and therapy, Szeged, Hungary; 2 Department of Physiology, Anatomy and Neuroscience, University of Szeged, Szeged, Hungary; 3 Hungarian Center of Excellence for Molecular Medicine Research Group for Cancer Genomics and Epigenetics, Szeged, Hungary; 4 Doctoral School of Experimental and Preventive Medicine, University of Szeged, Szeged, Hungary; 5 ELKH-SZTE Research Group for Cortical Microcircuits, Department of Physiology, Anatomy and Neuroscience, University of Szeged, Szeged, Hungary; 6 European Molecular Biology Laboratory, Heidelberg, Germany; 7 Department of Optics and Quantum Electronics, University of Szeged, Szeged, Hungary; 8 Hungarian Centre of Excellence for Molecular Medicine Research Group for Translational Medicine Development, Szeged, Hungary; 9 Department of Neurosurgery, University of Debrecen Clinical Centre, Debrecen, Hungary; 10 Department of Neurosurgery, Borsod County Hospital, Miskolc, Hungary; 11 Department of Neurosurgery, University of Szeged, Szeged, Hungary; 12 Neuronal Cell Biology Research Group, Eötvös Loránd University, Budapest, Hungary

**Keywords:** Ion channels in the nervous system, Cellular neuroscience

## Abstract

Human cortical interneurons differ from their rodent counterparts in intrinsic membrane properties, yet the mechanisms regulating excitability across physiologically relevant membrane potentials remain poorly defined. Here, we investigated inwardly rectifying potassium (Kir) channel control of subthreshold excitability in parvalbumin-expressing (Pvalb) interneurons from human and mouse neocortex. Using whole-cell recordings, dynamic clamp, patch sequencing, immunofluorescence, and computational modeling, we show that membrane hyperpolarization induces a proportional decrease in input resistance mediated by Kir channels in both species, despite higher baseline input resistance in human neurons. Transcriptomic and anatomical analyses revealed somatic membrane expression of four major Kir channel subtypes with moderate interspecies differences. Kir activation suppresses intrinsic excitability through combined voltage-dependent and shunting inhibition, an effect occurring during inhibitory postsynaptic potentials evoked by neurogliaform cells. Together, these findings show that homologous Pvalb neurons in humans have evolved toward a conserved, archetypal excitability phenotype, despite substantial differences in baseline excitability between species.

## Abstract (web summary)

Kir potassium channels proportionally suppress subthreshold excitability in human and mouse Pvalb interneurons, revealing a conserved mechanism that stabilizes cortical circuit function despite marked interspecies differences in baseline membrane properties.

## Introduction

Rodents are widely used as model organisms in neurobiological research. However, rodent neurons differ from human neurons, limiting the utility of rodent cells for clarifying the molecular and functional characteristics of neurons in the human brain. Human brain tissue, obtained during surgery, serves as a valuable resource for these studies.

Even homologous neuron types—defined by shared anatomical location, morphology, developmental origin, and marker gene expression—exhibit interspecies differences in gene and protein expression profiles across mammals^1,2^. Studies using ex vivo human cortical slices have revealed functional distinctions between human and rodent^3,4^. The species characteristics of neuron types are best understood in the neocortex, a component of the cortical mantle involved in complex brain functions^5–7^.

One prominent difference between human and rodent neurons concerns input excitability, commonly assessed by membrane input resistance (Rin). Compared with mouse neurons, human neocortical pyramidal neurons show reduced responsiveness to current injection due to lower Rin^8–11^, whereas human inhibitory interneurons exhibit enhanced responsiveness associate with higher Rin^12–16^. However, most previous studies have measured Rin near resting membrane potentials (approximately −60 mV), leaving its regulation across broader, physiologically relevant voltage ranges largely unexplored.

Parvalbumin-expressing (Pvalb) interneurons—the largest inhibitory population in the cortical mantle^17–19^—display higher Rin in humans than in rodents^14,15,20^. These fast-spiking interneurons mediate rapid γ-aminobutyric acid (GABA)ergic inhibition and are broadly conserved across species, sharing molecular markers (e.g., *PVALB, KCNC1, KCNC2*) and electrophysiological hallmarks such as brief action potentials and modest firing accommodation^21–25^. Nonetheless, differences in gene expression, ion channel distribution, and intrinsic electrophysiological properties between human and rodent Pvalb neurons have been reported^14,15,20,21,26–30^.

In rodents, ion channel mechanisms governing input resistance and intrinsic excitability across subthreshold membrane potentials are well characterized^31–36^. In particular, inwardly rectifying potassium (Kir) channels play a central role by activating at subthreshold voltages to suppress excitatory inputs^30,31,34^. By contrast, the contribution of Kir channels to excitability regulation in human Pvalb neurons remains poorly understood.

To address this gap, we investigated Kir channel–mediated regulation of subthreshold excitability in human and mouse Pvalb neurons using whole-cell electrophysiology, dynamic clamp, patch sequencing, immunofluorescence imaging, and computational modeling. We show that Kir channels exert a quantitatively similar inhibitory influence in both species, despite marked differences in baseline membrane resistance. Kir-mediated inhibition operates at resting membrane potentials and is progressively enhanced during membrane hyperpolarization, producing potent shunting inhibition of excitatory inputs. Molecular analyses confirmed the expression of KCNJ3 (Kir3.1), KCNJ6 (Kir3.2), KCNJ9 (Kir3.3), and KCNJ4 (Kir2.3) in Pvalb neurons, while confocal immunofluorescence revealed subtype-specific differences in somatic membrane localization between human and mouse neurons. Functionally, Kir channel activation during inhibitory synaptic events provides strong suppression of excitatory input in the soma of human Pvalb neurons.

## Results

**Fig. 1:**
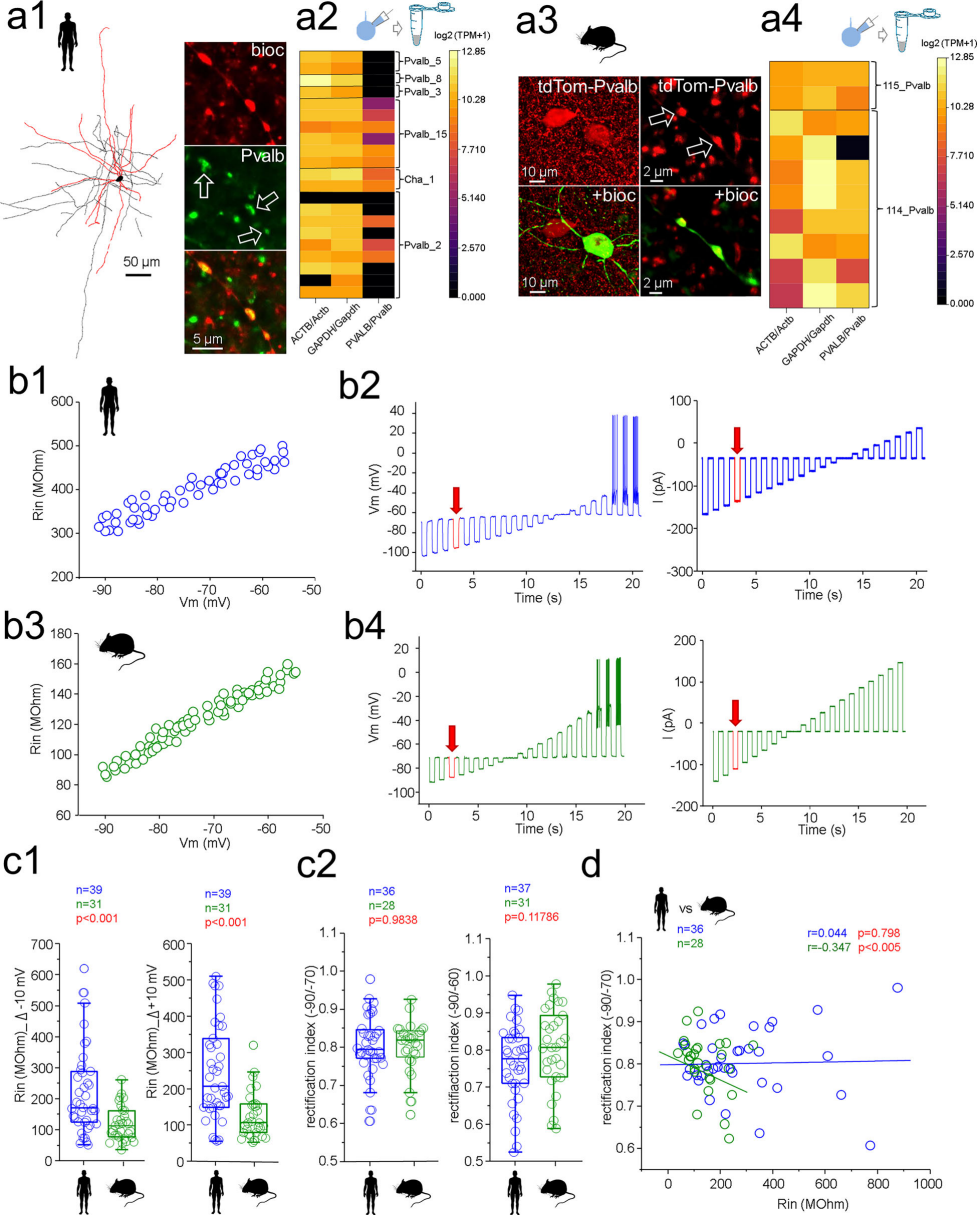
Proportional decreases in input excitability during membrane hyperpolarization are conserved between human and mouse parvalbumin (Pvalb) neurons despite differences in Rin. **a** Identification of Pvalb interneurons in neocortical layer 2/3. (a1) Human Pvalb neurons: recorded cells were filled with biocytin (bioc) for *post hoc* visualization using fluorophore-conjugated streptavidin and analyzed for Pvalb expression by fluorescence immunohistochemistry. Left: biocytin-filled human Pvalb neuron in a 60 μM section (dendrite and soma red, axon black). Right: confocal images showing Pvalb-positive axon boutons (pointed by arrows) of the same cell. (a2) Alternatively, recorded cells were analyzed by patch-sequencing. Six Pvalb neuron subtypes were identified using the Allen Institute Neocortical Neuron Type Identification System; some cells showed undetectable *PVALB* mRNA. (Cell i.d. codes shown in right). (a3) Mouse Pvalb neurons: identified by tdTomato (tdTom) fluorescence in genetically labeled Pvalb cells. Confocal images show tdTom-positive axon boutons by arrows. (a4) Patch-sequencing analysis revealed two mouse Pvalb subtypes (Allen Institute classification, i.d. codes shown in the right). Nearly all cells (9/10) expressed *PVALB* mRNA. **b** Current–voltage (I–V) relationships in human and mouse Pvalb neurons. Incremental current steps revealed nonlinear subthreshold I–V relationships. All experiments were performed in the presence of the HCN channel blocker ZD7288 (50 μM). (b1) Rin at different Vm values in a human Pvalb neuron. (b2) Voltage responses to hyperpolarizing and depolarizing current pulses (500 ms) from a resting Vm of −70 mV. One −100 pA step is highlighted in red (arrow). (b3) Rin at different Vm values in a mouse Pvalb neuron. (b4) Voltage responses to current pulses in a mouse neuron; a − 100 pA step is highlighted in red (arrow). **c** Human and mouse Pvalb neurons exhibit comparable proportional decreases in Rin during hyperpolarization, despite human neurons having higher absolute Rin. All experiments included ZD7288 (50 μM). (c1) Rin measured with −10 mV (left) or +10 mV (right) voltage steps in human and mouse Pvalb neurons. (c2) Input resistance ratio (r_Rin_) calculated as Rin at −90 mV / Rin at −70 mV (left) and Rin at −90 mV / Rin at −60 mV (right). Ratios were similar in human and mouse neurons. Permanova and *post hoc* Mann-whitney pairwise test with Bonferroni corrections for repetitive measures. **d** Relationship between rRin and Rin. In human neurons, rRin was not correlated with Rin, whereas in mouse neurons a negative correlation was observed (Spearmann Rank Order correlation, *p*-value with t-statistics). The data underlying this Figure can be found in Supplementary Data.

We studied human Pvalb neurons (*n* = 39; see Supplementary Table 1 for cell and patient details) identified by either immunohistochemistry (Fig. 1a1) or transcriptomic analysis (Fig. 1a2). Neurons were recorded in neocortical slices obtained from neurosurgical resections. Eighteen neurons were confirmed as Pvalb-positive by immunostaining after intracellular biocytin filling. Twenty-one cells were identified using patch-seq. Eleven cells exhibited detectable levels of *PVALB* mRNA. For the remaining ten patch-sequenced neurons in which *PVALB* transcripts were undetectable, cell identity was assigned using the Allen Institute Neocortical Neuron Type Classification System (https://knowledge.brain-map.org/mapmycells/process/), which classifies layer 2/3 neocortical neuron types based on their global gene expression profiles. This assigned these cells to types characterized by very low *PVALB* expression (Fig. 1a2, Supplementary Table 2)^37^. In parallel, mouse Pvalb neurons were examined in *PVALB*-tdTomato reporter mice, where tdTomato fluorescence labels *PVALB*-expressing cells (*n* = 31; Fig. 1a3–4).

### Input resistance similarly drops by membrane potential hyperpolarization in human and mouse Pvalb neurons

We found that both human and mouse Pvalb neurons exhibited a decrease in input resistance (Rin) in response to membrane hyperpolarization from resting potential. To quantify this, we applied square-pulse current steps (250–500 ms) at −70 mV in whole-cell current-clamp mode to depolarize and hyperpolarize the membrane potential (Vm) and systematically measured somatic Rin at −90, −80, −70 and −60 mV (Fig. 1b1-4). As shown in Fig. 1c1, Rin values at all tested Vm levels were higher in human neurons (*n* = 36 cells) compared to mouse cells (*n* = 31 cells): −90 mV: 186.5 (127.0−305.1) MΩ vs. 95.7 (71.8−145.2) MΩ, *p* < 0.001; −80 mV: 194.3 (136.2−362.4) MΩ vs. 111.2 (81.5−180.9) MΩ, *p* = 0.007; −70 mV; 230.7 (162.4−364.7) MΩ vs. 113.9 (71.3−161.5) MΩ; −60 mV: 233.1 (170.4−391.2) MΩ vs. 124.0 (80.5−198.4) MΩ, *p* < 0.001 (Permanova and *post hoc* Mann-whitney pairwise test with Bonferroni corrections for repetitive measures) (Fig. 1c1). Despite these differences in absolute Rin, both species displayed a comparable proportional decrease in Rin during hyperpolarization The resulting input resistance ratios (r_Rin_) were similar in the two species at −90 mV/−70 mV (human = 0.795, 0.772−0.848; mouse = 0.818, 0770−0.843; *p* = 0.978, *n* = 39 and 31) (Fig. 1c1) as well as at −90 mV/−60 mV (human = 0.778, 0.710−0.840; mouse = 0.807, 0.727−0.893; *p* = 0.116, *n* = 37 and 31) (Permanova and *post hoc* Mann-whitney pairwise test with Bonferroni corrections for repetitive measures) (Fig. 1c2). r_Rin_ in human Pvalb neurons did not differ between male (*n* = 24 cells) and female (*n* = 12 cells) subjects at −90 mV/−70 mV (*p* = 0.0880) or at −90 mV/−60 mV (*p* = 0.570) (Mann–Whitney U test) (see Supplementary Table 1). All experiments were conducted in the presence of the HCN channel blocker ZD7288 (50 μM) to isolate Kir channel–mediated effects on Rin across hyperpolarized and depolarized Vm values^15^. The resistance ratio (measured as −90 mV/−70 mV) was not correlated with baseline Rin (measured at −60 mV) in human cells (*r* = 0.044, *p* = 0.797; *n* = 36), whereas in mouse cells a significant negative correlation was observed (*r* = −0.378, *p* = 0.049; *n* = 28; Fig. 1d) (Spearmann Rank Order correlation, *p*-value with t-statstics). This species difference may reflect the greater variability and wider range of baseline input resistance in human compared with mouse cells^38^, potentially arising from increased diversity of Pvalb interneuron subtypes in the human neocortex^1,39^.

### Voltage-dependent regulation of somatic input resistance is mediated by Kir-type potassium channels in human and mouse Pvalb neurons

**Fig. 2:**
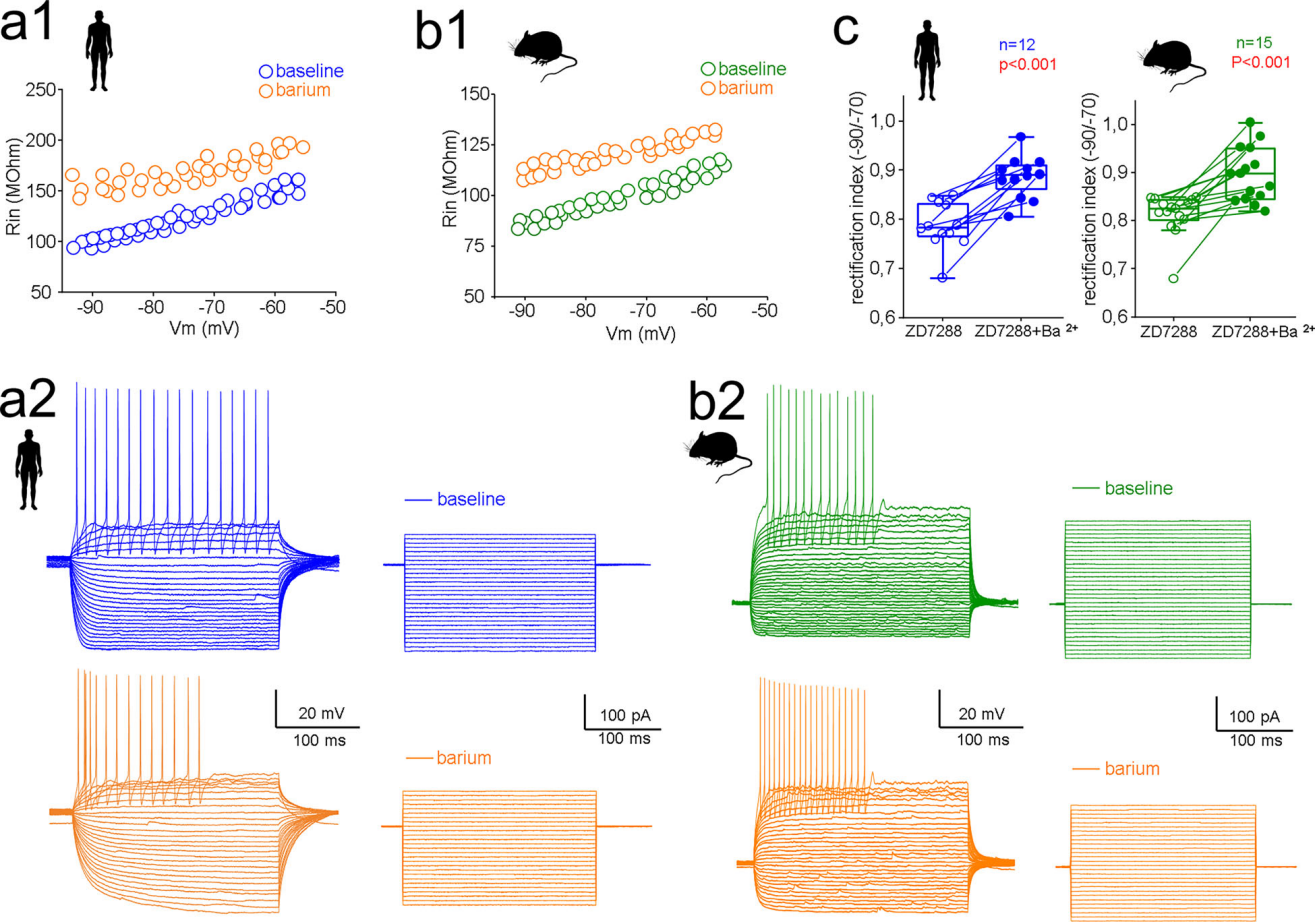
Ba²⁺ similarly reduces the proportional decrease in input resistance (Rin) in human and mouse parvalbumin neurons. Extracellular Ba²⁺ (100 μM) suppressed the hyperpolarization-dependent reduction in Rin. All experiments were performed in the presence of the HCN channel blocker ZD7288 (50 μM). (a1) Rin measured at different membrane potentials (Vm) in a human Pvalb neuron under control conditions (blue) and after 10 min Ba²⁺ application (orange). (a2) Representative Vm responses to current steps in control (blue) and Ba²⁺ (orange). (b1) Rin measured at different Vm values in a mouse Pvalb neuron under control and Ba²⁺ conditions. (b2) Representative Vm responses under both conditions. **c** Input resistance ratio (r_Rin_ measured as Rin at –90 mV / Rin at –70 mV) in human (blue) and mouse (green) Pvalb neurons before and after Ba²⁺ exposure. Ba²⁺ significantly increased r_Rin_ in both species (mixed-design ANOVA, *post hoc* comparisons with Wilcoxon signed-rank test). Box plots show the median, quartiles and 95th percentile. The data underlying this Figure can be found in Supplementary Data.

We next examined the mechanism underlying the voltage-dependent decrease in input resistance during membrane hyperpolarization. Bath application of Ba²⁺ (100 µM), a broad-spectrum blocker of Kir channels, markedly reduced the voltage dependence of Rin in both human (Fig. 2a1–2) and mouse Pvalb neurons (Fig. 2b1–2). In the presence of Ba²⁺, the progressive decrease in Rin at increasingly hyperpolarized membrane potentials (Vm) was attenuated, resulting in a more linear Rin–Vm relationship between −90 and −70 mV (Fig. 2c). Accordingly, the r_Rin_ ratio (Rin at −90 mV / Rin at −70 mV) in human Pvalb neurons increased from 0.78 (IQR 0.76–0.83) under control conditions to 0.89 (IQR 0.85–0.91) following Ba²⁺ application (*p* < 0.001, *n* = 12). Similarly, rRin in mouse Pvalb neurons increased from 0.83 (IQR 0.80–0.84) to 0.90 (IQR 0.85–0.95) after Ba²⁺ treatment (*p* < 0.001, *n* = 15; Wilcoxon signed-rank test; Fig. 2c1–2). As summarized in Table 1, Ba²⁺ produced a robust increase in r_Rin_ across species (main effect of time, *p* < 0.001; mixed-design ANOVA), with no significant interaction between species and treatment (interaction *p* = 0.51), indicating that the magnitude of the Ba²⁺ effect did not differ between human and mouse neurons. *Post hoc* comparisons were performed using Wilcoxon signed-rank tests within species and Mann–Whitney U tests between species at corresponding time points. Together, these results demonstrate that the voltage-dependent proportional reduction in Rin during membrane hyperpolarization is mediated by inwardly rectifying potassium (Kir) channel activation in both human and mouse Pvalb neurons^31,40,41^.

**Table 1:** Ba²⁺ similarly abolishes voltage-dependent input resistance rectification in human and mouse Pvalb neurons

	Human *n* = 12		Mouse *n* = 15		*P*-value				
	Baseline median, (IQR)	Ba^2+^, median, (IQR)	Baseline median, (IQR)	Ba^2+^, median, (IQR)	Mixed design ANOVA	Human (from baseline to Ba^2+^)	Mouse (from baseline to Ba^2+^)	Between human and mouse in baseline	Between human and mouse (in Ba^2+^)
rRin (-90 mV/-70 mV)	0.785 (0.763 to 0.834)	0.891 (0.853 to 0.913)	0.826 (0.803 to 0.844)	0.898 (0.846 to 0.952)	species: 0.113 time: < 10⁻⁶ interaction: 0.51	0.00049	6.1 × 10⁻⁵	0.113	0.788

The Kir channel blocker Ba²⁺ (100 µM) significantly reduces the hyperpolarization-induced decrease in input resistance in both human and mouse Pvalb neurons. Rectification ratios (r_Rin_), expressed as median and interquartile range (Q1–Q3), were calculated as Rin at −90 mV relative to −70 mV under control conditions and after Ba²⁺ application. Mixed-design ANOVA revealed significant main effect of time (baseline vs. Ba²⁺, human and mouse data pooled) indicating a consistent reduction in Rin rectification following Kir channel blockade across species. The species × time interaction *p*-value (*p* > 0.05) shows that the magnitude of the Ba²⁺ effect is similar between human and mouse neurons. *Post hoc* comparisons were performed using Wilcoxon signed-rank tests for within-species comparisons and Mann–Whitney U tests for between-species comparisons at corresponding time points.

### Human and mouse Pvalb neurons exhibit mRNA expression of Kir2.3, Kir3.1, Kir3.2 and Kir3.3 channels

**Fig. 3:**
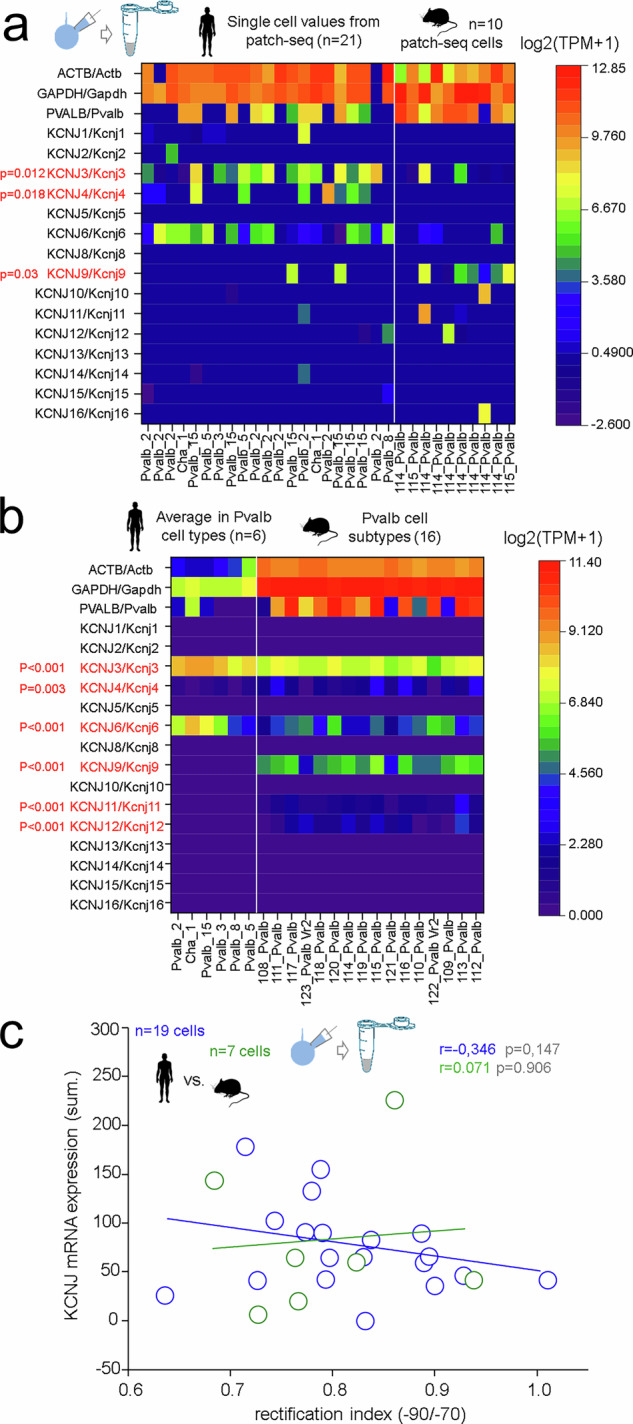
Human and mouse parvalbumin (Pvalb) neurons express Kir2.3, Kir3.1, Kir3.2 and Kir3.3 channel mRNA. **a** mRNA analysis in patch-sequenced human (*n* = 21) and mouse (*n* = 10) individual Pvalb type neurons. The heatmap shows transcripts per million (TPM) for *KCNJ1–16* (Kir family genes), and housekeeping genes *GAPDH* and *ACTB*. Pvalb cell identities were defined using the Allen Institute Neocortical Neuron Type Identification System (subtype codes on abscissa). Human and mouse neurons expressed *KCNJ3* (Kir3.1; higher in human, *p* = 0.012) and *KCNJ6* (Kir3.2), with higher *KCNJ9* (Kir3.3) in mouse (*p* = 0.03). Human cells additionally expressed *KCNJ4* (Kir2.3) which was undetected in the ten mouse cells. Left margin shows *p*-values for human–mouse comparisons (MANOVA with Bonferroni *post hoc* test); significant differences are in red. **b** Heatmap of averaged *KCNJ* gene expression (TPM) across Pvalb neuron subtypes in the temporal gyrus from the Allen Institute dataset. *KCNJ3* and *KCNJ6* expression is observed in all Pvalb neuron subtypes in both species; *KCNJ4, KCNJ9, KCNJ11*, and *KCNJ12* are higher in mouse (MANOVA with Bonferroni post hoc test). **c** Scatterplot showing no correlation between summed *KCNJ* mRNA levels (all subtypes) and input resistance ratio (rRin). rRin = 1 indicates no Kir effect (Spearmann Rank Order correlation, *p*-value with t-statstics). The data underlying this Figure can be found in Supplementary Data.

We analyzed the mRNA expression levels [transcripts per million (TPM)] of genes encoding Kir potassium channels in human and mouse Pvalb neurons in individual electrophysiologically recorded neurons using patch-sequencing^16^. Among the 16 KCNJ genes (*KCNJ1–16*) that encode Kir channel subunits, human neurons showed the highest expression of *KCNJ3* and *KCNJ6*, corresponding to Kir3.1 and Kir3.2 channels, respectively (Fig. 3a). Both *KCNJ3* and *KCNJ6* transcripts were also detected in mouse Pvalb neurons, though *KCNJ3* expression was significantly lower in mice (*p* = 0.012), whereas *KCNJ6* levels did not differ significantly between species (*p* = 0.07). Additional interspecies differences were observed for *KCNJ4* (Kir2.3), which was abundant in human but undetectable in mouse neurons (*p *= 0.018), and for *KCNJ9* (Kir3.3), which was robustly expressed in mouse but weakly expressed in human cells (*p* = 0.03). (MANOVA with Bonferroni *post hoc* test).

We next examined average transcript abundance (TPM) of *KCNJ* genes in transcriptomically defined Pvalb neuron subtypes from the human middle temporal gyrus, using datasets from the Allen Brain Institute^7,39^. Consistent with our patch-sequencing results from individual neurons, both *KCNJ3* (Kir3.1) and *KCNJ6* (Kir3.2) showed high average expression in Pvalb neuron types in both humans and mice (Fig. 3b), with significantly higher expression in humans than in mice (both p < 0.001). *KCNJ9* (Kir3.3) was robustly expressed in mouse Pvalb neuron types but was undetectable in humans (*p* < 0.001), closely matching our patch-sequencing findings. In contrast, *KCNJ4* (Kir2.3) showed strong average expression in mouse but low expression in human Pvalb subtypes (*p* < 0.001), differing from the results obtained from individual-neuron patch sequencing. Finally, the Allen dataset revealed higher average expression of *KCNJ11* and *KCNJ12* in mice than in humans (both *p* < 0.001), a species difference not observed in our single-cell patch-sequencing analysis. (MANOVA with Bonferroni *post hoc* test).

The total mRNA expression of Kir-type channels in the patch-sequenced Pvalb neurons (sum of TPM values for all *KCNJ* transcripts per cell) was not correlated with the magnitude of the hyperpolarization-induced decrease in Rin in either humans (*r* = 0.346, *p* = 0.147) or mice (*r* = 0.071, *p* = 0.906; Fig. 3c). Likewise, the total *KCNJ* mRNA level (summed TPM) showed no association with the absolute Rin measured at −70 mV (human: *r* = −0.384, *p* = 0.105; mouse: *r* =−0.178, *p* = 0.713). However, cytoplasmic *KCNJ* mRNA levels do not necessarily predict the abundance or subcellular localization of Kir proteins in the somatic membrane, as transcript levels captured at the time of nuclear collection may poorly reflect cellular protein abundance, likely due to burst-like rather than steady-state gene expression^42^. (Spearmann Rank Order correlation, *p*-value with t-statstics).

### Immunofluorescence reveals somatic membrane expression of Kir channels in Pvalb neurons across species

Therefore we conducted immunofluorescence analysis of the four expressed Kir channel types, namely Kir2.3, Kir3.1, Kir3.2 and Kir3.3 in human and mouse neocortical Pvalb cells. Human neurons exhibited Pvalb immunofluorescence within the soma (*n* = 50 cells collected from 3 human objects, 10 cells per reaction), whereas mouse cells demonstrated somatic emission of tdTomato (*n* = 50 cells from 3 animal sources, 10 cells in each reaction) driven by the *PVALB* promoter. These signals were used to identify the intracellular compartment, with the outer fluorescence boundary defining the somatic membrane region for subsequent analyses. We used two parallel analyses to investigate whether the Kir channels were localized in soma membrane.

**Fig. 4:**
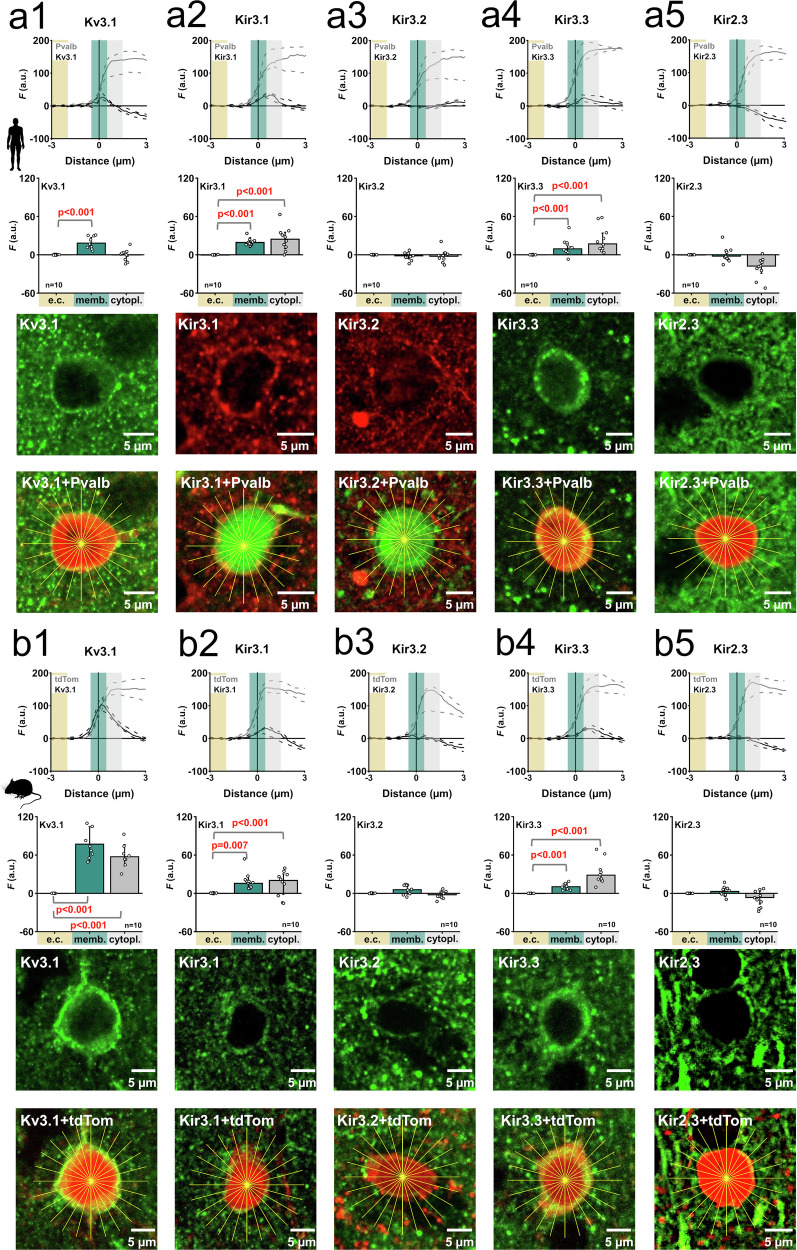
Immunofluorescence line-scan analysis reveals somatic membrane localization of Kir channels in human and mouse parvalbumin neurons. Immunofluorescence analysis (green, Alexa488; red, cy3 or tdTomato) combined with radial line-scan intensity measurements was used to examine the subcellular distribution of Kv3.1 and four Kir channel subtypes (Kir3.1, Kir3.2, Kir3.3, and Kir2.3) in human and mouse neocortical parvalbumin (Pvalb) neurons. Channel immunoreactivity was analyzed in parallel with Pvalb labeling in human tissue or *PVALB*-driven tdTomato fluorescence in mouse tissue to define somatic compartments. This approach enabled quantitative assessment of channel localization in the extracellular space, somatic membrane, and cytoplasm. (a1) Kv3.1 in human Pvalb neurons. *Top:* Representative fluorescence intensity (*F*) profiles for Pvalb (gray) and Kv3.1 (black) measured along 24 radial lines extending from the extracellular space (negative abscissa values) into the soma (positive values), crossing the extracellular membrane (0 ± 0.5 µm; green shaded region). The membrane location was defined by the abrupt onset of Pvalb fluorescence (see Methods). Solid lines indicate medians and dashed lines quartiles (Q1–Q3) across the 24 lines from a single representative cell. Extracellular fluorescence (yellow background) average was subtracted from all values. The cytoplasmic region is indicated by the gray shaded area. *Middle:* Summary of Kv3.1 fluorescence intensity across compartments (extracellular, membrane, cytoplasm) for 10 human Pvalb neurons. Kv3.1 is significantly enriched in the membrane (*p* < 0.001; ANOVA on ranks with Student–Newman–Keuls test). *Bottom:* Representative confocal image showing Kv3.1 immunofluorescence and Pvalb labeling, with overlaid radial line pattern used for intensity measurements. (a2–a5) Kir channels in human Pvalb neurons (*n* = 10 cells per condition). Kir3.1 (a2) and Kir3.3 (a4) show significant enrichment in the somatic membrane and cytoplasm (*p* < 0.001). In contrast, Kir3.2 (a3) and Kir2.3 (a5) do not show significant enrichment in either compartment. Bottom images illustrate KIR immunoreactivities. (b1) Kv3.1 in mouse Pvalb neurons. *Top:* Representative fluorescence intensity profiles (median and Q1-Q3) for tdTomato (gray) and Kv3.1 (black) measured along 24 radial lines in a single mouse Pvalb neuron. *Middle:* Quantification across 10 cells shows Kv3.1 enrichment in both the membrane and cytoplasm (*p* < 0.001; ANOVA on ranks with Student–Newman–Keuls test). *Bottom:* Confocal image showing Kv3.1 immunoreactivity, tdTomato fluorescence, and merged channels, with radial line overlays. (b2–b5) Kir channels in mouse Pvalb neurons (*n* = 10 cells per condition). Kir3.1 (b2) and Kir3.3 (b4) are significantly enriched in the somatic membrane and cytoplasm (Kir3.1: membrane *p* = 0.007, cytoplasm *p* < 0.001; Kir3.3: *p* < 0.001). Kir3.2 (b3) and Kir2.3 (b5) do not show consistent enrichment in either compartment, although occasional cells display weak somatic Kir2.3 signal (bottom images in b5). Together, these analyses demonstrate robust and conserved somatic membrane localization of Kir3.1 and Kir3.3 channels in human and mouse Pvalb neurons, whereas Kir3.2 and Kir2.3 exhibit low and variable somatic expression. The data underlying this Figure can be found in Supplementary Data.

We performed line-scan analyses of Kir and Pvalb (or tdTomato) fluorescence in confocal images. In each cell, fluorescence was measured along 24 radially oriented lines (acquisition at 5.5–15.5 pixels/µm). Line profiles quantified Pvalb (or tdTomato in mice) and Kir2.3, Kir3.1, Kir3.2, or Kir3.3 fluorescence along a 6-µm line extending from the extracellular space, across the membrane, and into the soma (Fig. 4). For each cell, the mean fluorescence intensity of pixels in the extracellular region was used as a reference (Fig. 4a–b) and subtracted from all pixels along the line profiles. Line profiles from individual cells were then averaged and are shown as median values with quartiles (Fig. 4a1). In this analysis, the extracellular membrane appeared as a narrow zone in which intracellular marker fluorescence rose sharply, as observed in both human Pvalb neurons (Fig. 4a1–5) and mouse tdTomato-labeled neurons (Fig. 4b1–5)^15^. To precisely define membrane localization, we performed parallel immunolabeling for Kv3.1, a potassium channel robustly expressed in Pvalb neurons. The membrane region was identified by the peak of Kv3.1 fluorescence, which consistently coincided with the onset of Pvalb or tdTomato signal (Fig. 4a1, b1). Based on this alignment, a 1-µm-wide membrane zone was defined from the intracellular fluorescence boundary and applied uniformly across all cells and conditions. Fluorescence intensities of Kir3.1 (Fig. 4a2, b2), Kir3.2 (Fig. 4a3, b3), Kir3.3 (Fig. 4a4, b4), and Kir2.3 (Fig. 4a5, b5) were then quantified in this membrane boundary. Kv3.1 line-scan analysis confirmed membrane enrichment in human Pvalb neurons (*p* < 0.001) with no detectable intracellular accumulation compared with extracellular levels (*p* = 0.734, *n* = 10. ANOVA on ranks with Student–Newman–Keuls test; Fig. 4a1). Analysis of Kir channels revealed significant enrichment of Kir3.1 and Kir3.3 at the somatic membrane of human Pvalb neurons compared with extracellular background (Kir3.1, *n* = 10, *p* < 0.001; Kir3.3, *n* = 10, *p* < 0.001), as well as significant intracellular signal for both channels (Kir3.1, *p* < 0.001; Kir3.3, *p* < 0.001; ANOVA on ranks with Student–Newman–Keuls test). In contrast, Kir3.2 (*n* = 10) and Kir2.3 (*n* = 10) showed no significant enrichment in either membrane or intracellular compartments relative to extracellular levels. These results are shown in Fig. 4a2–a5 for Kir3.1, Kir3.2, Kir3.3, and Kir2.3, respectively. In mouse Pvalb neurons, Kv3.1 was similarly enriched at the membrane (*n* = 10, *p* < 0.001) but also showed significant intracellular signal (*p* < 0.001; Fig. 4b1). Kir channel localization followed a comparable pattern: Kir3.1 and Kir3.3 were significantly enriched both at the membrane (Kir3.1, *n* = 10, *p* = 0.007; Kir3.3, *n* = 10, *p* < 0.001) and intracellularly (both *p* < 0.001). In contrast, neither Kir3.2 nor Kir2.3 exhibited significant membrane or intracellular enrichment (Kir3.2, *n* = 10, *p* = 0.089; Kir2.3, *n* = 10, *p* = 0.064). Mouse data are shown in Fig. 4b2–b5 for Kir3.1, Kir3.2, Kir3.3, and Kir2.3, respectively. Together, these data indicate that two Kir channel types are expressed at the somatic membrane in human and mouse Pvalb neurons. By contrast, Kir3.2 and Kir2.3 showed no consistent enrichment in somatic membrane or cytoplasmic compartments relative to extracellular background, indicating low somatic expression and/or predominant signal arising from surrounding neurites and glial elements.

**Fig. 5:**
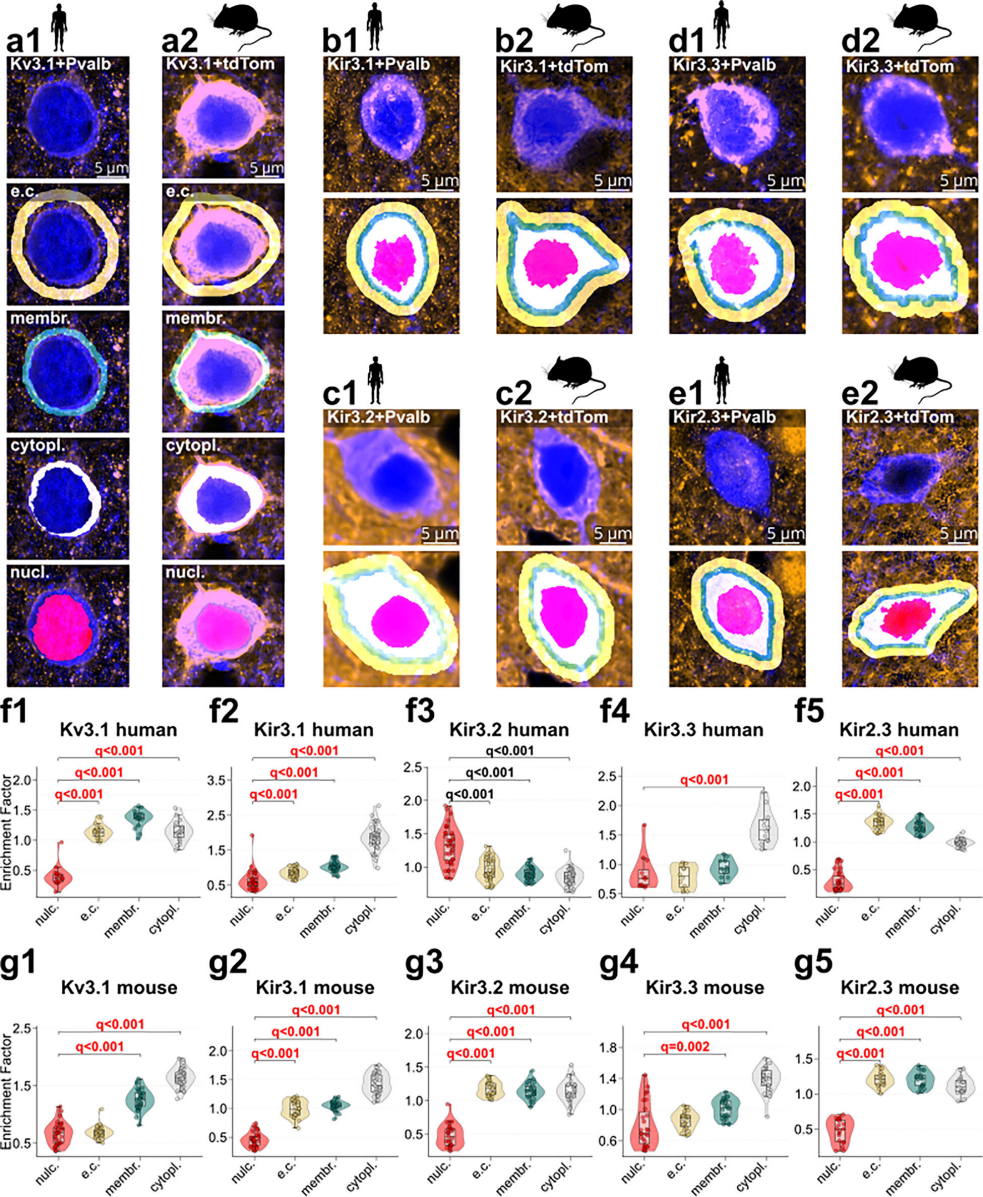
Image segmentation–based analysis reveals compartment-specific enrichment patterns of Kv3.1 and Kir channels in human and mouse parvalbumin neurons. Kv3.1 and Kir channel immunofluorescence was analyzed together with Pvalb labeling in human tissue or Pvalb-driven tdTomato fluorescence in mouse tissue using an automated segmentation-and-zoning pipeline. Compartments are: an extracellular annulus (e.c.) and a membrane-proximal belt (membr.), cytoplasmic zone (cytopl.) and a nuclear/core region (nucl.). The same colour coding is used for the segmented regions in the confocal images and the data plots. a1–a2 Example segmentation for Kv3.1 in human (a1) and mouse (a2) Pvalb neurons. From top to bottom, panels show the raw two-color visualization (Kv3.1; Pvalb or tdTomato) and the corresponding compartment overlays (e.c., membr., cytopl., nucl.). Color rendering is for display only and does not affect quantification. (b1–b2) Kir3.1, (c1–c2) Kir3.2, (d1–d2) Kir3.3, and (e1–e2) Kir2.3 example segmentations in human (1) and mouse (2) neurons. **f–g** Quantification of compartmental fluorescence for Kv3.1, and Kir channels expressed as enrichment factor (EF; Methods), normalizing signal to compartment area (EF = Signal%/Area%). Violin plots show EF distributions across analyzed cells/optical sections (numbers indicated above each panel). Values are compared against nucleus zone EF. Human Pvalb neurons showed enrichment in the membrane for (f1) Kv3.1, (f2) Kir3.1 and (f5) Kir2.3, but not to Kir3.2 or Kir3.3 (f3 and f4). (g1-g5) Mouse neurons showed enrichent of Kv3.1 and all four Kir channels in the membrane and cytoplasm. (Omnibus Kruskal–Wallis test across compartments followed by *post hoc* two-sided Mann–Whitney U tests versus the nuclear/core reference with Benjamini–Hochberg correction; FDR-adjusted *q*-values are shown above comparisons). In the violin plots, *q*-values are shown in red when the median EF of the compared compartment exceeds the reference median and in black when it is lower. EF = 1 corresponds to the optical-section–wide average intensity, EF > 1 indicates relative enrichment, and EF < 1 indicates relative depletion. The data underlying this Figure can be found in Supplementary Data.

In addition, we performed an independent quantitative analysis of Kv3.1 (Fig. 5a1–a2) and Kir channel immunofluorescence (Fig. 5b–e) in Pvalb neurons from human cortex and tdTomato-labeled Pvalb neurons from mouse cortex using a custom automated segmentation-and-zoning pipeline (ZoneSig) (https://github.com/HCEMM/ZoneSig). This approach enables unbiased identification of the soma and nucleus, which serves as an internal reference for quantifying ion-channel immunofluorescence in the cytoplasmic and somatic membrane compartments, and allows compartment-wide analysis. Somatic masks were generated from the Pvalb (human) or tdTomato (mouse) channel using the Cellpose–SAM model^43^ and used to define concentric perisomatic and intracellular regions. Specifically, extracellular, membrane, cytoplasmic, and nuclear compartments were delineated (Fig. 5a–e), and ion-channel signal was quantified within each compartment. Analyses were performed on the same immunostained tissue sets used for the line-scan experiments. For each neuron, confocal z-stacks were acquired and multiple optical sections were quantified, yielding per-section measurements that were summarized as enrichment factors (EF; Methods). EF provides a zone-normalized metric relating the fraction of ion-channel signal in a given compartment to the fractional area of that compartment, enabling direct comparison across cells and species (Fig. 5f–g). The intracellular compartment was further subdivided into cytoplasmic and nuclear regions using an unsupervised spectral clustering step prior to quantification (Fig. 5a–e) (https://github.com/HCEMM/ZoneSig). Across both human and mouse datasets, EF profiles revealed compartmental differences in Kir channel immunofluorescence. In human Pvalb neurons, Kv3.1 (*n* = 57 stacks of 19 cells from 3 human objects), Kir3.1 (*n* = 111 stacks of 37 cells, 3 human objects), and Kir2.3 (*n* = 84 stacks of 28 cells, 3 human objects), showed significant enrichment at the somatic membrane relative to the nucleus, whereas Kir3.3 ((*n* = 36 stacks of 12 cells, 3 human objects) was enriched in the cytoplasm but not in the membrane (Fig. 5f1–5). Kir3.2 (*n* = 120 stacks of 40 cells, 3 human objects), did not show significant enrichment in any compartment. *q*-values with statistical analysis are shown in detail in Fig. 5f. In mouse Pvalb neurons (Fig. 5g1–5), Kv3.1 (*n* = 108 stacks of 36 cells, 3 mice), and all four Kir channels were significantly enriched in the membrane, and all channels also showed cytoplasmic enrichment (Kir3.1, *n* = 90 stacks of 30 cells, 3 mice; Kir3.2 = 81 stacks of 27 cells, 3 mice; Kir3.3 = 81 stacks of 27 cells, 3 mice; Kir2.3 = 57 stacks of 19 cells, 3 mice) (Fig. 5g2–5). *q*-values for mouse are shown in detail in Fig. 5g. Enrichment was assessed using an omnibus Kruskal–Wallis test across compartments, followed by *post hoc* two-sided Mann–Whitney U tests comparing each compartment to the nuclear reference, with Benjamini–Hochberg correction for multiple comparisons. FDR-adjusted q values are reported in Fig. 5f–g.

### Computational modeling reveals input resistance regulation with lower Kir conductance in human than in mouse Pvalb neurons

**Fig. 6:**
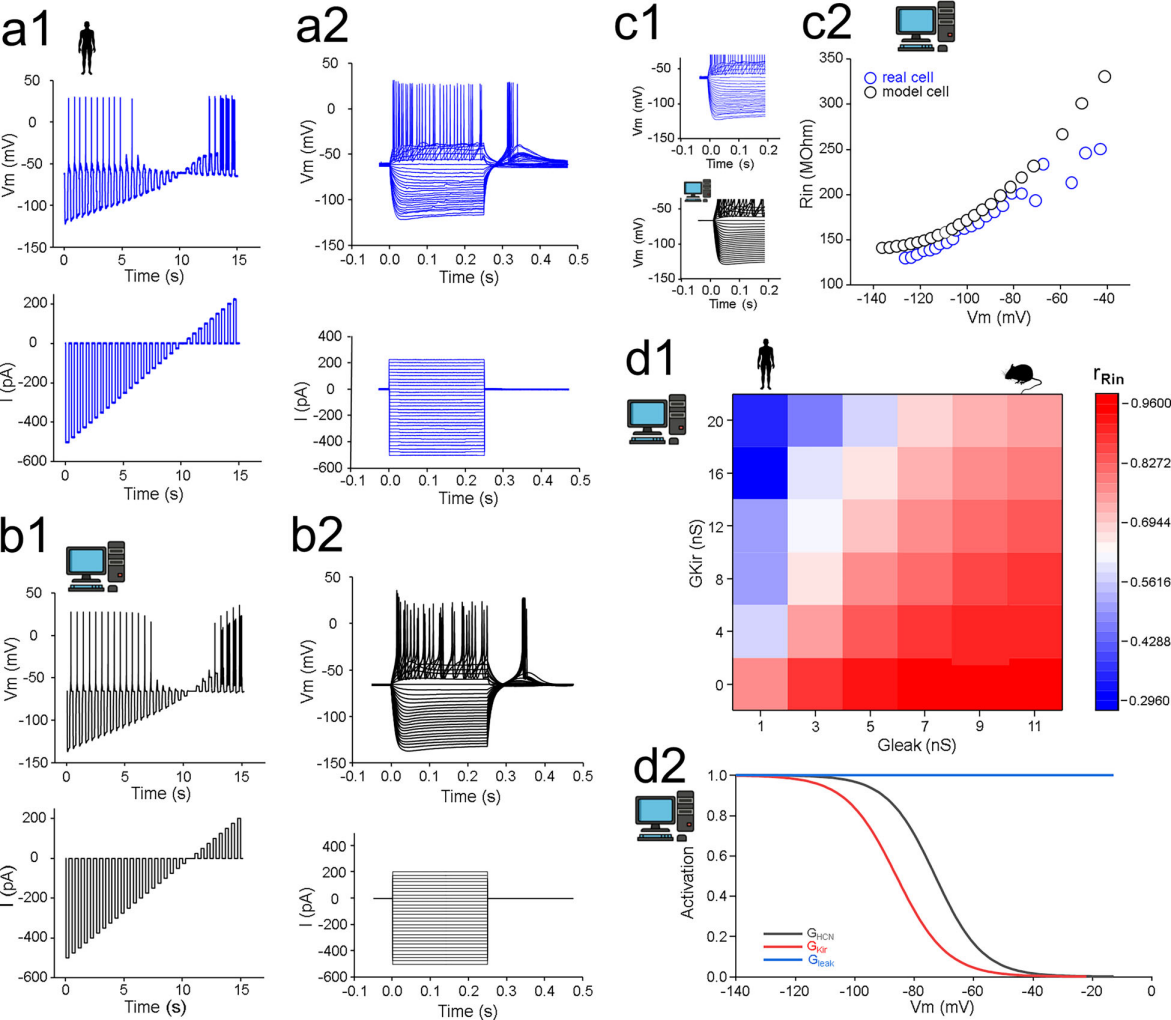
Computational modeling demonstrates input resistance rectification with lower Kir channel density in human than in mouse Pvalb neurons. **a** Whole-cell recording from a human neocortical parvalbumin (Pvalb) neuron. (a1) Current–voltage (I–V) step protocol showing membrane potential (Vm) responses to a series of current injections. (a2) Superimposed Vm responses (top) and corresponding current steps (bottom). Single-cell computational model incorporating passive and voltage-dependent conductances in the somatic membrane reproduces the Vm responses of the recorded neuron. (b1) Simulated I–V step protocol. (b2) Superimposed simulated Vm responses (top) and injected current steps (bottom). **c** The model reproduces the voltage dependence of input resistance (Rin) observed experimentally. (c1) Superimposed Vm responses from the recorded human Pvalb neuron (blue) and the model neuron (black). (c2) Rin measured at different membrane potentials in the real neuron (blue) and in the model (black). **d** Dependence of somatic input resistance rectification on leak conductance (G_leak_) and Kir conductance (G_Kir_) in the model. The input resistance ratio (rRin) was calculated from I–V steps as Rin at −90 mV divided by Rin at −70 mV. Simulations were performed using low G_leak_ values characteristic of human Pvalb neurons and higher G_leak_ values characteristic of mouse neurons (abscissa; symbols on top indicate the direction of human and mouse cell type ends of the range of somatic G_leak_ values), across a range of G_Kir_ values (ordinate). A small HCN conductance was included (G_HCN_ = 0.4 nS at −70 mV and 0.9 nS at −90 mV), consistent with experimental observations^15^. (d1) Heat map showing rRin values for different combinations of G_leak_ (1–11 nS) and G_Kir_ (0–20 nS). (d2) Voltage dependence of model conductances. G_Kir_ is voltage-dependent, reaching ~10% of its maximal activation at −70 mV and ~60% at −90 mV (red line). G_leak_ is voltage-independent (blue line). The HCN conductance (black line) has a maximal value of 1 nS, with steady-state activation ranging from 0.4 to 0.9 nS over the illustrated voltage range. The data underlying this Figure can be found in Supplementary Data.

We used a single-cell computational model to reproduce the membrane potential (Vm) dynamics of Pvalb neurons recorded in whole-cell configuration. A representative Pvalb neuron (neuron H4; Supplementary Table 1) with intrinsic electrophysiological properties common to both human and mouse cells was selected as the experimental reference (Fig. 6a1–2). The model neuron (Fig. 6b1–2) consisted of a soma containing passive leak conductance (Gleak), HCN-type currents, Kv7- and Kv1-type potassium currents in the soma and dendrites, and action potential–generating Na⁺ currents in the axon^44^. Model input resistance and membrane capacitance were matched to experimental values. Somatic current injections (−300 to +200 pA, 250 ms) produced membrane potential responses that closely matched those of the recorded neuron (Fig. 6c1–2). To determine the Kir conductance required to reproduce the experimentally observed input resistance ratio (rRin), the model was simulated across a range of somatic leak conductances (Gleak, 1–11 nS), spanning values observed in human (low Gleak) and mouse (high Gleak) Pvalb neurons at −70 mV. In parallel, maximal somatic Kir conductance (GKir_max_) was varied from 0 to 20 nS (Fig. 6d1). Because Kir conductance is voltage dependent, steady-state activation reached ~10% of GKir_max_ at −70 mV and ~60% at −90 mV (Fig. 6d2). These simulations show that neurons with lower Gleak, characteristic of human Pvalb neurons, require substantially less Kir conductance to reproduce the observed input resistance rectification than neurons with higher Gleak, characteristic of mouse Pvalb neurons. The steady-state voltage dependence of Gleak, Kir, and HCN conductances used in the model is shown in Fig. 6d2^15^.

### Regulation of input excitability in human Pvalb neurons during Kir-mediated hyperpolarization

**Fig. 7:**
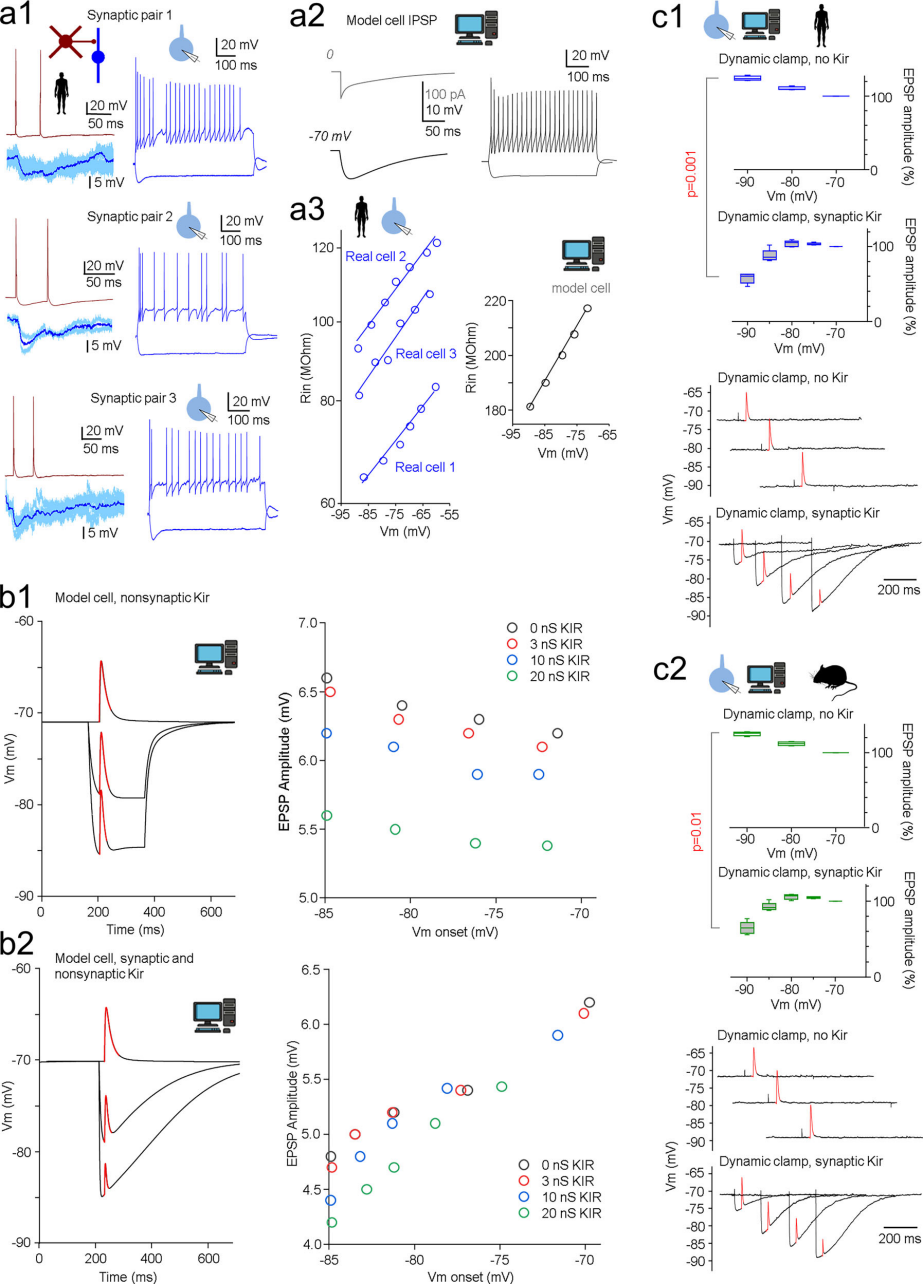
Kir-mediated synaptic inhibition suppresses input excitability during slow IPSPs in Pvalb neurons. Recordings from synaptically connected putative neurogliaform (NGF) cells and parvalbumin (Pvalb) interneurons reveal a physiological mechanism by which Kir channel activation reduces input excitability during slow inhibitory postsynaptic potentials (IPSPs). Slow IPSPs, mediated by GABA_B_ receptor–dependent activation of Kir channels^12,45–47^. **a** Real cell pair recordings showing slow IPSP in human fast-spiking interneurons. Whole-cell paired recordings (cells 1–3) from monosynaptically connected presynaptic putative NGF neurons (layer 1 soma) and postsynaptic fast-spiking interneurons (layer 2/3 soma) in human neocortex. *Left*, presynaptic NGF action potentials (brown; two 5-ms depolarizing pulses separated by 50 ms) evoke slow IPSPs in postsynaptic Pvalb neurons (blue; bold trace indicates mean). *Inset*, schematic of recording configuration. *Right*, representative Vm responses of the postsynaptic fast-spiking neuron to depolarizing and hyperpolarizing square-pulse current steps. (a2) Single-compartment Pvalb neuron model reproducing slow IPSPs evoked by NGF input. *Left*, schematic of the simulated IPSP (gray, injected synaptic current; black, membrane potential). *Right*, Vm behavior of the model neuron during square-pulse current steps. (a3) Membrane hyperpolarization reduces Rin in experimentally recorded Pvalb neurons (blue) and in the computational model (gray), as measured using current-step protocols in the absence of IPSPs. **b** Model-based analysis of Kir-mediated suppression of excitatory postsynaptic potentials (EPSPs, red). (b1) Non-synaptic Kir activation during membrane hyperpolarization induced by square-pulse current injection. *Left:*Simulated EPSPs evoked by fixed-amplitude EPSCs (3 nS) injected at the soma at resting membrane potential (−70 mV) and during hyperpolarization (maximum somatic GKir = 10 nS). *Right:* EPSP amplitude (measured from onset to peak) plotted as a function of membrane potential during current-step hyperpolarization. Somatic G_Kir_ was varied from 0 to 20 nS (color-coded). (b2) Combined non-synaptic and synaptic Kir activation. EPSPs were paired with synaptic Kir-mediated IPSPs of increasing amplitude (maximum synaptic G_Kir_ up to 420 nS), while somatic (non-synaptic) G_Kir_ was varied between 0 and 20 nS (colour coded). Simulations show progressive suppression of EPSP amplitude with increasing Kir-mediated hyperpolarization and a synergistic inhibitory effect of synaptic and non-synaptic Kir conductances. **c** Dynamic-clamp validation of Kir-mediated EPSP suppression. Dynamic-clamp experiments were performed while endogenous Kir channels were blocked with Ba²⁺ (100 µM), isolating the effect of synaptic dynamic clamp-mediated Kir-mediated conductances. (c1) Human Pvalb neurons (*n* = 5). Top, EPSPs evoked at around −70, −80, and −90 mV using hyperpolarizing current steps under Ba²⁺ did not differ significantly in amplitude (*p* = 0.86; ZD7288, 50 µM present). Bottom, dynamic-clamp–generated Kir-mediated IPSPs significantly reduced EPSP amplitude at −90 mV compared with EPSPs evoked at the same membrane potential without Kir activation. Traces show EPSPs (red) at distinct Vm. (**c2**) Mouse Pvalb neurons (*n* = 4). Top, EPSPs evoked at different membrane potentials using current steps. Bottom, EPSPs evoked during dynamic-clamp–generated Kir-mediated IPSPs. Kir-mediated IPSPs significantly suppress EPSP amplitude at −90 mV in both species (ANOVA on ranks with Dunn’s post hoc test). Traces show representative recordings. Summary data are shown as median and quartiles. The data underlying this Figure can be found in Supplementary Data.

In cortical circuits, slow inhibitory postsynaptic potentials (IPSPs) generated by neurogliaform (NGF) cells activate postsynaptic Kir channels through GABA_B_ receptors in fast-spiking interneurons, producing prolonged hyperpolarization and a pronounced decrease in input resistance (Rin)^12,45–50^. We recorded from synaptically paired putative NGF and Pvalb neurons in the human neocortex (*n* = 3 pairs; Fig. 7a1). Single or paired action potentials in NGF cells reliably evoked slow IPSPs (>150 ms) in postsynaptic Pvalb neurons. IPSPs with comparable kinetics were reproduced in the Pvalb neuron model that accurately captured the membrane properties and firing behavior of the recorded cells (Fig. 7a2–3)^12,45–48^.

Using this model, EPSPs were simulated under three conditions: (i) membrane hyperpolarization induced by current injection via the recording pipette while varying somatic nonsynaptic Kir conductance (G_Kir_), (ii) identical levels of hyperpolarization with Kir channels blocked (Fig. 7b1), and (iii) during Kir-mediated synaptic IPSPs (Fig. 7b2). HCN channels were absent in the simulations. Activation of nonsynaptic Kir channels in the somatic membrane during current-induced hyperpolarization (G_Kir_  = 0–20 nS; see also Fig. 6c–d) reduced EPSP amplitude evoked by a fixed EPSC conductance (3 nS) at all membrane potentials compared with conditions lacking Kir conductance (Fig. 7b1). EPSP suppression was substantially stronger during synaptic Kir-mediated IPSPs (IPSC conductance up to 46 nS), with pronounced inhibition already observed for IPSPs of ~10 mV (Fig. 7b2), comparable to those evoked in human Pvalb neurons by single NGF cell connections (Fig. 7a1^51^. Notably, synaptic Kir-mediated inhibition of EPSPs was most pronounced when non-synaptic somatic Kir conductance was also present (G_Kir_ = 3–20 nS), indicating synergistic suppression of input excitability by synaptic and extrasynaptic Kir activation.

This prediction was tested experimentally using dynamic clamp to impose Kir-mediated IPSPs in human and mouse Pvalb neurons while endogenous Kir channels were blocked with Ba²⁺ (100 µM). EPSPs were generated simultaneously with Kir-mediated IPSPs of increasing amplitude to quantify EPSP suppression. As a reference condition, EPSCs of fixed conductance (3 nS) were injected at different membrane potentials set by square current pulses (−70, −80, and −90 mV) while endogenous Kir channels were blocked with Ba^2+^ (100 μM). Under these conditions, hyperpolarization alone did not significantly alter EPSP amplitude in either species, despite the increased electrochemical driving force for EPSCs (Fig. 7c1–2)^31,50^. In human neurons (*n* = 5), EPSP amplitudes were 7.41 mV (IQR 6.81–8.67) at −70 mV, 8.06 mV (IQR 7.61–9.62) at −80 mV (*p* = 1.00), and 8.98 mV (IQR 8.70–10.70) at −90 mV (p = 0.86). In mouse neurons (*n* = 4), EPSP amplitudes were 6.90 mV (IQR 7.41–8.46) at −70 mV, 7.76 mV (IQR 7.62–9.26) at −80 mV (*p* = 1.00), and 8.68 mV (IQR 8.53–10.35) at −90 mV (*p* = 1.00; ANOVA on ranks with Dunn’s *post hoc* test). All experiments were performed in the presence of the HCN channel blocker ZD7288 (50 μM)^15^. In contrast, Kir-mediated IPSPs imposed by dynamic clamp under blockade of the neuron’s endogenous Kir channels robustly suppressed EPSP amplitude at hyperpolarized membrane potentials. At −90 mV, EPSP amplitude was reduced to 4.52 mV (IQR 4.29–5.83) in human Pvalb neurons (*p* = 0.001 vs. EPSP at −90 mV without IPSP; *n* = 5) and to 5.47 mV (IQR 4.83–6.23) in mouse neurons (*p* = 0.01; *n* = 4).

Thus, Kir-mediated postsynaptic inhibition produces a robust suppression of excitatory input, demonstrating a powerful physiological mechanism for regulating input excitability in Pvalb neurons across species.

## Discussion

Understanding how intrinsic membrane mechanisms regulate neuronal excitability across physiologically relevant membrane potentials is not well understood in human neurons. Here, we demonstrate that inwardly rectifying potassium (Kir) channels exert a conserved and powerful control over subthreshold excitability in parvalbumin-expressing (Pvalb) interneurons of both the human and mouse neocortex. Despite pronounced species differences in baseline input resistance (Rin), Kir channel activation produces a remarkably similar proportional reduction in Rin and suppression of excitability across species, with moderate differences in the soma membrane Kir channel composition. These findings identify Kir-mediated inhibition as an archetypal and evolutionarily conserved mechanism that stabilizes Pvalb neuron activity in mammalian cortical circuits.

Human Pvalb interneurons exhibit substantially higher Rin than their rodent counterparts, consistent with previous reports of reduced passive membrane leak in human inhibitory neurons. Nonetheless, membrane hyperpolarization elicited a proportional decrease in Rin that was quantitatively similar in humans and mice^50^. This voltage-dependent rectification was abolished by micromolar Ba²⁺, identifying Kir channels as the principal mediators of this effect. Thus, Kir channels scale their inhibitory influence relative to the cell’s baseline electrical properties.

Computational modeling revealed that human Pvalb neurons require substantially lower somatic Kir conductance to reproduce the experimentally observed Rin rectification compared with mouse neurons. This difference arises from the higher baseline Rin of human neurons and suggests a form of biophysical efficiency: comparable functional inhibition can be achieved with fewer active Kir channels. Such “neuroeconomic” scaling may reduce the metabolic burden associated with maintaining ionic gradients in fast-spiking interneurons, which are among the most energetically demanding cell types in the cortex^52,53^. This principle contrasts with other conductances, such as HCN or Kv1 channels, whose density and localization differ in Pvalb neurons markedly between species, highlighting Kir channels as a conserved regulator of interneuron excitability^15,16,38^.

Kir channels influence excitability through two complementary mechanisms: voltage-dependent hyperpolarization and shunt inhibition. Both mechanisms were evident in our experimental and modeling data. Somatic Kir activation reduced Rin progressively with membrane hyperpolarization, thereby dampening the impact of excitatory inputs even as the driving force for excitatory synaptic currents increased^31,51,53,54^. Importantly, this effect was not reproduced by hyperpolarization alone when Kir channels were blocked, demonstrating that Kir conductance is required for EPSP suppression. Kir activity rapidly suppresses input–output action potential (AP) generation, and protects Pvalb neurons from excessive discharges^55–57^.

This inhibitory role became particularly evident during slow inhibitory postsynaptic potentials (IPSPs) mediated by neurogliaform (NGF) cells. NGF-evoked GABA_B_ receptor activation recruits Kir channels, generating prolonged hyperpolarization accompanied by a pronounced decrease in Rin. Dynamic clamp experiments and simulations showed that such Kir-mediated IPSPs strongly suppress EPSPs, with substantial inhibition already at ~10 mV hyperpolarization—well within the physiological range produced by single NGF connections in human cortex. Notably, EPSP suppression was strongest when synaptic Kir activation occurred in the presence of extrasynaptic Kir conductance, indicating synergistic inhibition by synaptic and non-synaptic Kir pools. Although Kir-mediated inhibition was robust in both species, we observed species-specific variability. In humans, Pvalb neurons with either high or low Rin could exhibit strong or weak Kir-mediated inhibition, whereas in mice the magnitude of Kir inhibition correlated with Rin. This difference may reflect greater genetic and transcriptomic diversity among Pvalb subtypes in the human compared with the mouse neocortex^7,39^. Consistent with this interpretation, Kir gene expression varies across Pvalb neuron types between species.

Our transcriptomic and immunohistochemical analyses indicate that multiple Kir channel subtypes contribute to this conserved physiological function^31^. Patch sequencing and Allen Institute datasets consistently identified Kir3.1 and Kir3.2 as predominant transcripts in Pvalb neurons across species, with additional contributions from Kir3.3 and Kir2.3 that differed between humans and mice. Although single-cell mRNA levels were not found to correlate with Kir-mediated rectification, this is not unexpected given the complex regulation of mRNA expression, which may occur in bursts, and the targeting of channel proteins to the membrane.

Immunofluorescence analyses revealed somatic membrane localization of Kir channels in both species, although the precise subtype distribution differed depending on the analytical approach. Line-scan analyses suggested membrane enrichment of Kir3.1 and Kir3.3, whereas segmentation-based compartment analysis indicated prominent membrane localization of Kir3.1 and Kir2.3 in human neurons and broader membrane expression of all examined Kir subtypes in mice. Discrepancies between mRNA abundance and protein localization—particularly for Kir3.3 in human neurons—may reflect burst-like transcription, post-transcriptional regulation, or technical limitations in antibody specificity, which remain challenging to resolve in human tissue lacking genetic knockout controls. Nonetheless, all approaches converge on the conclusion that somatic Kir channels are positioned to exert strong control over synaptic integration in Pvalb neurons and that human cells do not systematically show, or show very low level of, Kir3.2 in the soma membrane.

Mouse Pvalb neurons rely on multiple Kir subtypes for flexible inhibition in fast circuits. Human Pvalb neurons achieve the required level of inward rectification and gain control with fewer Kir subtypes indicating that additional molecular diversity offers limited incremental benefit. The reduced Kir subtype diversity in human Pvalb neurons may reflect functional optimization rather than limitation. Given the larger size, higher input resistance, and distinct scaling properties of human neurons, proportional Kir-mediated gain control can be achieved with fewer molecular components with less molecular and pharmacological diversity. Such simplification may represent a neuroeconomic strategy that minimizes metabolic cost while preserving function with energy-efficient circuit design. This aligns with broader principles of large brain energy optimization.

Functionally, Kir channels place Pvalb neurons under powerful inhibitory control by slow synaptic inputs^12,45^. NGF cells are ideally suited to engage this mechanism: they generate widespread, slow GABA_B_ receptor–mediated inhibition that recruits Kir channels and suppresses interneuron excitability over hundreds of milliseconds^12,46,47,58^. Through this pathway, NGFs can transiently disengage Pvalb-mediated fast inhibition, potentially facilitating transitions between cortical network states such as active processing, quiescence, and slow-wave activity^51^.

Beyond GABA_B_ receptors, Kir channels are coupled to multiple neuromodulatory receptors, including opioid and monoaminergic receptors, linking this mechanism to behavioral states, pain processing, mood regulation, and the actions of addictive drugs^30,49,59,60^. The conservation of Kir-mediated inhibition across species therefore supports the translational relevance of rodent models for understanding modulatory control of human cortical interneurons, while also highlighting quantitative differences that may influence drug sensitivity.

This study has limitations that should be considered. Inter-individual variability in human tissue, including differences in age and cortical region, may contribute to the broader range of intrinsic biophysical properties observed in human neurons. In addition, larger and more homogeneous cohorts will enable clearer Kir channel subtype-specific analyses in the future.

Systematic identification of the same Pvalb neurons by both patch-sequencing and post hoc immunohistochemistry is methodologically challenging in acute human slice experiments, particularly after prolonged whole-cell recordings. Therefore, neurons were classified using either transcriptomic profiling or immunolabeling. Importantly, both approaches are well established: patch-sequencing–based identification relied on full-transcriptome analysis and classification using the Allen Institute cell-type framework, which is widely considered a robust method for defining Pvalb neuron identity.

Kir channel function was assessed primarily using Ba²⁺, a broad-spectrum Kir blocker, which demonstrates Kir dependence but does not resolve the relative contributions of individual subtypes; more selective pharmacological or genetic approaches—currently not feasible in acute human tissue—would allow more definitive attribution of function to specific Kir channels. In addition, HCN channels were blocked using micromolar concentrations of ZD7288 to isolate Kir-mediated effects. While this approach is standard, off-target effects on other conductances cannot be fully excluded. However, any such nonspecific actions are unlikely to alter the central conclusion of the study, as Kir-mediated rectification and its proportional similarity between species were consistently observed under identical experimental conditions in both human and mouse neurons.

Likewise, patch-sequencing captures mRNA at a single time point and may miss transiently or lowly expressed genes, and immunofluorescence analyses remain constrained by antibody specificity and the absence of genetically validated knockout controls in human material. Electrophysiological recordings were performed in acute slices under partially reduced network conditions and in the presence of HCN channel blockade to isolate Kir effects; while necessary for mechanistic clarity, these conditions may not fully replicate in vivo neuromodulatory tone and intact circuit dynamics. Finally, computational modeling relied on a simplified single-cell framework with predominantly somatic Kir conductance, and synaptically paired and dynamic-clamp datasets were modest in size, limiting the resolution of spatial and subtype-specific effects. Future studies combining larger human datasets, Kir channel subtype-selective manipulation, genetically validated molecular tools, and more comprehensive single-cell computational models will be important to further refine species-specific mechanisms and circuit-level implications of Kir-mediated regulation.

In summary, our study identifies Kir-mediated regulation of excitability as a defining and conserved property of Pvalb interneurons in the mammalian neocortex. Despite substantial species differences in baseline membrane resistance and Kir channel subtype expression, the proportional inhibitory impact of Kir activation on synaptic integration is strikingly similar in human and mouse neurons. By combining voltage-dependent hyperpolarization with powerful shunt inhibition, Kir channels provide an efficient and robust mechanism for stabilizing Pvalb neuron output and shaping cortical network dynamics. These findings establish Kir channels as central regulators of interneuron function and underscore their importance for understanding both normal cortical computation and its dysregulation in neurological and psychiatric disease.

## Methods

### Ethics statement

Participant recruitment and inclusion procedures complied with the conditions of the approved ethics licenses. Written informed consent was obtained from all patients prior to surgery. For participants under 18 years of age, consent was obtained from a parent or legal guardian. All animal procedures were approved by the Governmental Office of Animal Health and Welfare (permit CS/I01/03036-2/2024) and the University of Szeged Ethics Committee. Human studies were approved by the Regional Human Investigation Review Board (reference 75/2014) and the National Scientific and Research Ethics Committee (ETT TUKEB; license BM/25042-1/2024). All procedures conformed to the principles of the Declaration of Helsinki.

### Human brain slice preparation

Human neocortical slices were prepared from tissue obtained during neurosurgical resections targeting deep brain structures. Tissue originated from frontal, temporal, or other cortical regions; patient demographics and cortical locations are provided in Supplementary Table 1. Anesthesia was induced with intravenous midazolam (0.03 mg/kg) and fentanyl (1–2 μg/kg) following a bolus intravenous injection of propofol (1–2 mg/kg). Additionally, patients received 0.5 mg/kg rocuronium to facilitate endotracheal intubation. During surgery, patients were ventilated with a 1:2 mixture of O_2_/N_2_O, and anesthesia was maintained with sevoflurane. Following surgery, the resected tissue blocks were immediately immersed in an ice-cold solution containing (in mM) 130 NaCl, 3.5 KCl, 1 NaH_2_PO_4_, 24 NaHCO_3_, 1 CaCl_2_, 3 MgSO_4_, and 10 d-(+)-glucose aerated with 95% O_2_/5% CO_2_ within a glass container on ice inside a thermally isolated box (within 20 min) and transported from the operating room to the electrophysiology laboratory with continuous 95% O_2_/5% CO_2_ aeration. Slices (350 μm) were cut using a vibrating microtome (Microm HM 650 V, Thermo Fisher Scientific) and incubated at 22–24 °C for 1 h. During incubation, slicing solution was gradually exchanged for recording solution (circulating volume 180 mL; 6–7 mL/min). The recording solution was identical to the slicing solution except for the addition of 3 mM CaCl₂ and 1 mM MgSO₄.

### Mouse brain slice preparation

Transverse cortical slices (350 μm) were prepared from the somatosensory and frontal cortices of 5–12-week-old heterozygous B6.129P2-Pvalbtm1(cre)Arbr/J mice (stock 017320, B6 PVcre line, Jackson Laboratory, Bar Harbor, ME, USA) crossed with the Ai9 reporter line, enabling tdTomato expression in Pvalb neurons for targeted recordings^15^. Both sexes were used.

### Whole-cell electrophysiology

Recordings were performed in a submerged chamber perfused with recording solution (8 mL/min) maintained at 36–37 °C. The cells were patched using visual guidance with infrared differential interference contrast video microscopy and a water immersion ×20 objective with an additional ×2-4 zoom. An appropriate dichroic mirror and filter (Cy3) was used for mouse tissue tdTomato signal epifluorescence. All recordings were completed within 30 min from entering the whole-cell mode. Micropipettes (5–7 MΩ) were filled with an intracellular solution with the following composition (in mM): 126 K-gluconate, 8 NaCl, 4 ATP-Mg, 0.3 Na_2_-GTP, 10 HEPES, and 10 phosphocreatine (pH 7.0–7.2; 300 mOsm) supplemented with 0.3% (w/v) biocytin for subsequent staining with fluorophore-conjugated streptavidin. Recordings were performed using a Multiclamp 700B amplifier (Axon Instruments, Scottsdale, AZ, USA). The recorded signal was low-pass–filtered online at a cutoff frequency of 6–8 kHz (Bessel filter). The series resistance and pipette capacitance were compensated in the current–clamp mode. All parameters were measured from at least five reproduced traces. Data were acquired using Clampex software (Axon Instruments), digitized at 35–50 kHz, and analyzed offline using NeuroExpress (version 24.c.02, developed by Attila Szucs)^15,61^, pClamp (version 10.5, Axon Instruments), Spike2 (version 8.1, Cambridge Electronic Design, Milton, UK), OriginPro (version 9.5, OriginLab Corporation, Northampton, MA, USA), and SigmaPlot (version 14, Grafiti, Palo Alto, CA, USA). Input resistance (Rin) was calculated as ΔVm/ΔI from steady-state membrane potentials reached during voltage steps, in the presence of ZD7288 (50 μM).

### Pharmacology

ZD7288 and BaCl_2_ (Sigma-Aldrich, St. Louis, MO, USA) were diluted in physiological extracellular solution and applied by bath perfusion.

### Visualization of biocytin-filled neurons

Cells filled with biocytin were visualized using either Alexa 488-conjugated streptavidin (1:1000, Jackson ImmunoResearch, West Grove, PA, USA) or Cy3-conjugated streptavidin (1:1000, Jackson ImmunoResearch). After recording, the slices were immediately fixed for at least 12 h in 4% paraformaldehyde and 15% picric acid in 0.1 M phosphate buffer (PB; pH 7.4) at 4 °C and then stored at 4 °C in 0.1 M PB containing 0.05% sodium azide as a preservative. All slices were embedded in 20% gelatin and further cut into 50–60-μm-thick sections in ice-cold PB using a vibratome (Microm HM 650 V). The sections were rinsed in 0.1 M PB (thrice for 10 min each), cryoprotected in 10–20% sucrose solution in 0.1 M PB, flash-frozen in liquid nitrogen, and thawed in 0.1 M PB. They were then incubated in 0.1 M Tris-buffered saline (TBS; pH 7.4) containing fluorophore-conjugated streptavidin for 2–3 h at 22–24 °C. After washing with 0.1 M PB (thrice for 10 min each), the sections were covered in Vectashield mounting medium (Vector Laboratories, Burlingame, CA, USA), placed under a coverslip, and examined under an epifluorescence microscope at ×20–60 magnification with the appropriate excitation light filters and dichroic mirrors (Leica DM 5000 B, Leica, Wetzlar, Germany)^15^.

For morphological reconstruction, selected sections were processed using avidin–biotin horseradish peroxidase and visualized with the glucose oxidase–DAB–nickel method (1:300; Vector Labs) in TBS (pH 7.4) at 4 °C. The enzymatic reaction was visualized by the glucose oxidase–diaminobenzidine–nickel method using 3,3′-diaminobenzidine tetrahydrochloride (0.05%) as the chromogen and 0.01% H_2_O_2_ as the oxidant. Sections were further treated with 1% OsO_4_ in 0.1 M PB. After several washes in distilled water, the sections were stained with 1% uranyl acetate, dehydrated in an ascending series of ethanol concentrations, infiltrated overnight with epoxy resin (Durcupan, Merck, Darmstadt, Germany), and embedded on glass slides. Light microscopic reconstructions were conducted using the Neurolucida system (MBF Bioscience, Williston, VT, USA) with a ×100 objective (Olympus BX51, Olympus UPlanFI, Olympus, Tokyo, Japan). Images were collapsed in the z-axis for illustration.

### Immunohistochemistry

Free-floating sections were washed in TBS containing 0.3% Triton X-100 (TBST) and blocked in 20% horse serum. Primary antibodies diluted in TBST were applied for three nights at 4 °C, followed by fluorophore-conjugated secondary antibodies overnight at 4 °C. Primary antibodies included goat anti-pv polyclonal (1:1000, PVG213, SWant, Switzerland, https://www.labome.com/product/SWant/PVG213.html); rabbit anti-Kv3.1 polyclonal (1:500, 242-0 P, Synaptic Systems, https://sysy.com/product/242003#list).; rabbit Anti-GIRK1 polyclonal (1:200, APC-005, https://www.alomone.com/p/anti-kir3-1-girk1/APC-005?srsltid=AfmBOoq8Ci8aXbBSAwiOBUMrXIjVvTu5etrTOGDtb2Jw948pwyvRUuB); rabbit Anti-GIRK2 antibody monoclonal (1:200, EPR23841-83, https://www.abcam.com/en-us/products/primary-antibodies/girk2-antibody-epr23841-83-ab259909); rabbit Anti-Kir3.3 polyclonal antibody (1:200, PA5-67106, https://www.thermofisher.com/antibody/product/Kir3-3-KCNJ9-Antibody-Polyclonal/PA5-67106); rabbit AntiKir2.3 polyclonal antibody (1:200, APC-032, https://www.alomone.com/p/anti-kir2-3/APC-032?srsltid=AfmBOoqN4Qb9PE2Dr8UxTqvk49bGopwPjQwLLTM_udmQCR8bhnUyGnu4). Secondary antibodies were: DARb Alexa 647–conjugated donkey anti-rabbit (1:200, Abcam, www.abcam.com); DAGt Cy3-conjugated donkey anti-goat (1:400, Jackson ImmunoResearch www.jacksonimmuno.com); DARb Cy3-conjugated donkey anti-rabbit (1:400, Jackson ImmunoResearch www.jacksonimmuno.com); DARb Alexae 488-conjugated donkey anti-rabbit (1:400, Jackson ImmunoResearch www.jacksonimmuno.com);

DAGt Alexa 488-conjugated donkey anti-goat (1:400, Jackson ImmunoResearch www.jacksonimmuno.com).

### Confocal imaging and immunofluorescence analysis

Brain slices labeled for immunofluorescence were acquired using a Leica Stellaris 8 laser-scanning confocal microscope (Nikon CFI Apo TIRF 100XC Oil, NA = 1.49) with 488, 554- and 649-nm lasers for excitation of the fluorophores. The utilized emission filters for these lasers were 480–535, 559–654 and 654–750 nm, respectively. Images were acquired using an HC PL APO CS2 ×63/1.40 oil immersion objective in the unidirectional scanning mode.

Line analysis of the immunofluorescence intensity was performed offline in confocal microscope images using LAS X Life Science Microscope Software (Leica) as described previously^15^. Images were analyzed using ImageJ (US National Institutes of Health, Bethesda, MD, USA). The intensity profiles of Kv3 and Kir and Pvalb/tdTomato fluorescence channels were acquired along each line from outside the cell to inside of the soma. To determine the edge and cytoplasm of the cell soma, the threshold of the Pvalb/tdTomato fluorescence signal (onset) was determined by using the IsoData thresholding method. The extracellular membrane location was marked as 0-point and determined by the Pvalb/tdTomato fluorescence signal onset [(threshold = average of the cell background pixels fluorescence subtracted from the average of the cell pixels fluorescence)/2]. The average intensity value of pixels in the extracellular area of each line (starting 0.5 µm from the Pvalb or TdTomato signal onset and extending away from the cell centre) was subtracted from each pixel’s intensity value. In the pooled analysis of lines, means and upper and lower quartiles were calculated for each position along the lines.

Compartment-resolved quantification of ion-channel immunofluorescence was performed using a custom Python pipeline (Python 3.12.3) with a Streamlit-based graphical user interface (GUI; streamlit 1.52.0), operating on individual confocal optical sections extracted from z-stacks (readlif 0.6.6). Somata were segmented from the Pvalb (human) or tdTomato (mouse) channel using the Cellpose–SAM model (Cellpose 4.0.8)^43^. Segmentation outputs were filtered to remove implausible detections (e.g., hollow objects and low-signal masks inconsistent with Pvalb/tdTomato-positive somata) (numpy 2.3.0, scipy 1.16.2, scikit-image 0.25.2)^62–64^. The final soma mask served as the geometric reference for deterministic compartment construction. Compartments were derived from the soma mask using morphological dilation and erosion with offsets expressed as fixed percentages of soma size (scikit-image 0.25.2, scipy 1.16.2). The extracellular compartment (e.c.) was defined as a concentric annulus outside the soma boundary spanning +10% to +40% outward dilation of the soma mask. The membrane-proximal compartment (membr.) was defined as a belt around the soma boundary computed as the set difference between the +10% dilated soma mask and the −10% eroded soma mask. The cytoplasmic compartment (cytopl.) was defined as the intracellular shell between −10% and −50% inward erosion of the soma mask, and the nuclear/core compartment (nucl./core) as the remaining deep interior after −50% erosion. In crowded fields, the extracellular annulus could be optionally cleaned by subtracting overlaps with neighboring somata (numpy 2.3.0). Where purely geometric separation was insufficient, the cytopl. versus nucl./core split could be refined using spectral clustering applied to pixels within the intracellular region, combining ion-channel intensity and radial position (distance to soma center) with tunable feature weights (intensity weight, radial weight) (scikit-learn 1.7.0, numpy 2.3.0). Ion-channel signal was quantified per optical section and compartment by aggregating pixel intensities within the compartment mask. Specifically, for compartment $c$, the area ${A}_{c}$ was defined as the number of pixels in the mask, and the signal ${S}_{c}$ as the sum of ion-channel intensities over those pixels (numpy 2.3.0). To enable robust cross-cell comparisons while accounting for differences in compartment size and overall staining intensity, compartmental enrichment was summarized as an enrichment factor (EF), defined as:

$${{EF}}_{c}=\,\frac{{Signal} \% }{{Area} \% }=\frac{({S}_{c}/\,{\sum }_{k}{S}_{k})}{({A}_{c}/{\sum }_{k}{A}_{k})}$$

where ${{\rm{Signal}}} \% \,$ is the fraction of the total ion-channel fluorescence signal contained within compartment $c$ (i.e., ${S}_{c}\,$ divided by the summed intensities ${\sum }_{k}{S}_{k}$ across all compartments ${k}$ of the same optical section), and ${{\rm{Area}}} \% \,$ is the fraction of the total compartment area occupied by compartment $c$ (i.e., ${A}_{c}\,$ divided by the total pixel count ${\sum }_{k}{A}_{k}\,$ across compartments within the same optical section). Under this normalization, ${EF}=1\,$ corresponds to the optical-section–wide average intensity, ${EF} > 1\,$ indicates relative enrichment of the ion-channel signal within that compartment, and ${EF} < 1\,$ indicates relative depletion (i.e., lower-than-average signal density) compared with the section-wide mean. Implementation details (software versions and full dependency list) are provided in the accompanying code repository (github.com/APPLICATION).

### Patch sequencing and transcriptomic analysis

Nuclear extraction, cDNA amplification, library preparation, and sequencing were performed as described previously^16^ using a Smart-seq2–based protocol^65,66^. Paired-end reads (66 bp) were aligned to GRCh38 (Ensembl GRCh38_r93) using STAR v2.7.11a^67^ with SmartSeq-specific parameters. TPM values derived from full gene counts were used for analysis.

### Identification of Pvalb neurons

Cells were classified using the Allen Institute Neocortical Neuron Type Identification System [Allen Institute for Brain Science, 2024, MapMyCells, available from https://knowledge.brain-map.org/mapmycells/process/ (RRID:SCR_024672)]. For human samples, the Seattle Alzheimer’s Disease Brain Cell Atlas reference taxonomy was used with the deep generative mapping algorithm, whereas mouse cells were classified using the 10X whole mouse brain reference taxonomy with the hierarchical mapping algorithm. In selected experiments, Pvalb identity was confirmed by immunofluorescence.

### Dynamic clamp

Dynamic clamp experiments were implemented using Signal software (Cambridge Electronic Design, Cambridge, UK) and a Power1401-3A interface. IPSPs and EPSPs were generated in real time based on membrane voltage. Kir-mediated slow IPSCs had a reversal potential of −95 mV and a peak conductance of up to 46 nS. EPSCs had a reversal potential of 0 mV, a time-to-peak of 0.3 ms and a decay time constant of 3 ms, with gEPSCs of 2–4 nS in human cells and 2–5 nS in mouse cells^38^.

### Computational modeling

Single-compartment neuron models were implemented in Delphi using Heun’s integration method (time step 1 μs)^61,68^. The soma model neuron was based on a previously described formalism^44,61^. The model featured a somatic compartment. G_leak_ of the somatic compartment in Fig.5 was 1.42 nS, G_Kir_ (maximum) was 10 nS (Fig. 6c1), and Cm of the somatic compartment was 80 pF. The leakage reversal potential was set to −68 mV. Model parameters were adjusted to reproduce experimental membrane responses. All intrinsic voltage-dependent currents were calculated as previously described^16^. The parameters of the synaptic IPSPs and EPSPs in the model were the same as those in the dynamic clamp experiments.

### Statistics and reproducibility

Data are presented as median and interquartile range. Statistical tests were run with Mann–Whitney U test, Wilcoxon test, Kruskal–Wallis ANOVA, MANOVA with Bonferroni post hoc test, Permanova test, mixed-design ANOVA, and Spearman rank correlations. Sample sizes are reported with *p*-values.

### Figures and artwork

The icons and schematic elements were modified from silhouette images adapted from the open-access source Wikimedia Commons (https://commons.wikimedia.org/). All other schematics were generated by the authors.

## Supplementary information

Transparent Peer Review file

Supplementary Information

Description of Additional Supplementary files

Supplementary Data

Reporting-summary

## Data Availability

Custom analysis scripts used in this work are available in a publicly accessible repository; (ZoneSig) (https://github.com/HCEMM/ZoneSig). Software versions and full dependency list are in the accompanying code repository (github.com/APPLICATION).

## Data availability

All data needed to evaluate the conclusions of this study are included in the paper and the Supplementary Materials, or have been uploaded as source files with the manuscript. Custom code used in this study is publicly available in a public repository (ZoneSig; https://github.com/HCEMM/ZoneSig) and in the accompanying code repository (github.com/APPLICATION). All other data are available from the corresponding author upon reasonable request.Source data supporting the findings of this study are provided with the figures in the manuscript.
