# Supplementary material for: Conserved Kir channel mechanisms governing intrinsic excitability in human and rodent parvalbumin neurons
Source: Commun Biol. 2026 Apr 13;9:806. doi: 10.1038/s42003-026-10063-9 (PMC13265741; doi:10.1038/s42003-026-10063-9)
Supplement: Supplementary file 2 — Supplementary Information [file 42003_2026_10063_MOESM2_ESM.pdf]

cell code	Patch-seq/IHC	sex	age (years)	hemisphere	region	diagnosis
h1	Patch-seq	M	13	right	frontal	tumor
h2	Patch-seq	M	13	right	frontal	tumor
h3	Patch-seq	M	13	right	frontal	tumor
h4	Patch-seq	M	13	right	frontal	tumor
h5	Patch-seq	M	13	right	frontal	tumor
h6	Patch-seq	M	59	right	frontal	tumor
h7	Patch-seq	M	59	right	frontal	tumor
h8	Patch-seq	M	NA	left	frontal	hydrocephal
h9	Patch-seq	M	NA	left	frontal	hydrocephal
h10	Patch-seq	M	NA	left	frontal	hydrocephal
h11	Patch-seq	M	NA	left	frontal	hydrocephal
h12	Patch-seq	F	51	right	insula	tumor
h13	Patch-seq	F	51	right	insula	tumor
h14	Patch-seq	F	51	right	insula	tumor
h15	Patch-seq	F	51	right	insula	tumor
h16	Patch-seq	F	51	right	insula	tumor
h17	Patch-seq	F	51	right	insula	tumor
h18	Patch-seq	F	51	right	insula	tumor
h19	Patch-seq	M	70	left	temporal	cavernoma
h20	Patch-seq	F	65	left	occipital	tumor
h21	Patch-seq	F	24	right	parietal	hydrocephal
h22	IHC	F	24	right	parietal	hydrocephal
h23	IHC	F	24	right	parietal	hydrocephal
h24	IHC	M	44	left	frontal	tumor
h25	IHC	M	44	left	frontal	tumor
h26	IHC	F	70	left	parietal	hydrocephal
h27	IHC	M	21	right	parietal	tumor
h28	IHC	M	21	right	parietal	tumor
h29	IHC	M	58	right	frontal	tumor
h30	IHC	M	58	right	frontal	tumor
h31	IHC	F	60	right	parietal	hydrocephalus
h32	IHC		36	right	frontal	hydrocephalus
h33	IHC	M	19	right	temporal	NA
h34	IHC	M	69	right	parietal	hydrocephal
h35	IHC	M	65	left	frontal	tumor
h36	IHC	F	58	left	parietal	tumor
h37	IHC	M	54	left	temporal	tumor
h38	IHC	NA	NA	NA	NA	NA
h39	IHC	NA	NA	NA	NA	NA

Supplementary Table 1. Details of human neocortical tissue resected during surgery, which was used to investigate the neurons in the study. From left to right: Cell code; Analysis method for parvalbumin (Pvalb) expression in the cells (IHC = immunohistochemistry; patch-seq = mRNA analysis); Patient sex (M = male, F = female) and age (years); Cerebral hemisphere (left or right); Cortical region; and Diagnosis for surgery.

human

cell ID	detected genes K	cluster label	bootstrapping p
TPM_A1_LOT5/ A150823_1	7,4	Pvalb_2	0,9999
TPM_A13_LO6/A220823_6	5,1	Pvalb_2	0,9998
TPM_A14_LOT6/A220823_7	5,4	Pvalb_2	1
TPM_A13 LOT10_A061023_1	7,1	Pvalb_2	1
TPM_A18 LOT10_A061023_6	7,2	Pvalb_2	0,7642
TPM_A19 LOT10_A061023_7	4,4	Pvalb_2	0,9968
TPM_A21 LOT10_A061023_9	8	Pvalb_2	0,9996
TPM_A5lot11_A261023_5	3,8	Pvalb_2	0,9999
TPM_A9 LOT09_A280923_6	3,9	Pvalb_2	0,9999
TPM_A16_LOT6/A220823_9/Cha	6,6	Chandelier_1	1
TPM_A22 LOT10_A061023_10/Cha	4,6	Chandelier_1	1
TPM_A4_LOT5/A150823_4	8,3	Pvalb_15	1
TPM_A11 LOT09_A280923_8	6,4	Pvalb_15	0,9982
TPM_A20 LOT10_A061023_8	8,3	Pvalb_15	0,9291
TPM_A6 LOT09_A280923_3	4,9	Pvalb_15	1
TPM_A7 LOT09_A280923_4	7,9	Pvalb_15	1
TPM_A8 LOT09_A280923_5	8	Pvalb_15	0,9998
TPM_A10 LOT09_A280923_7	4,2	Pvalb_3	0,9985
TPM_V11_V261023_3	6,9	Pvalb_8	0,9627
TPM_A9_LOT6/A220823_2	6,8	Pvalb_5	0,9997
TPM_A12 LOT09_A280923_9	7	Pvalb_5	1

mouse

cell ID	detected genes K	cluster label	bootstrapping p
TPM_M3_LOT1/A150224_3	3,2	114	1
TPM_M7_LOT1/A150224_7	3,7	114	1
TPM_M9_LOT1/A150224_9	3,5	114	1
TPM_M1_LOT2/A280224_1	4	114	1
TPM_M3_LOT2/A280224_3	6,8	114	1
TPM_M6_LOT2/A280224_6	5,5	114	1
TPM_M8_LOT2/A280224_8/OB	4,6	114	0,78
TPM_MV2_LOT2	4,5	114	1
TPM_MV5_LOT2	4	115	1
TPM_M5_LOT1/A150224_5	3,4	115	0,77

Supplementary Table 2. A table presenting the bootstrapped p-values for a specific single-cell mRNA sequencing database containing individual cells obtained by patch-sequencing. These cells were categorized according to the cell type classification system from the Allen database (https://knowledge.brain-map.org/mapmycells/process/) [39]. The values in the table were generated by the MapMyCells algorithm, which compares the entire transcriptomic profile of our cells with that of the published database. In addition, the table contains the detected genes for each cell, expressed in thousands (k).
