# Supplementary material for: Conserved Kir channel mechanisms governing intrinsic excitability in human and rodent parvalbumin neurons
Source: Commun Biol. 2026 Apr 13;9:806. doi: 10.1038/s42003-026-10063-9 (PMC13265741; doi:10.1038/s42003-026-10063-9)
Supplement: Supplementary file 5 — Reporting-summary [file 42003_2026_10063_MOESM5_ESM.pdf]

nature portfolio

Corresponding author(s): Karri Lamsa

Last updated by author(s): 20 jan 2026

# Reporting Summary

Nature Portfolio wishes to improve the reproducibility of the work that we publish. This form provides structure for consistency and transparency in reporting. For further information on Nature Portfolio policies, see our Editorial Policies and the Editorial Policy Checklist.

## Statistics

For all statistical analyses, confirm that the following items are present in the figure legend, table legend, main text, or Methods section.

| n/a | Confirmed | |
|---|---|---|
| ☐ | ☒ | The exact sample size (*n*) for each experimental group/condition, given as a discrete number and unit of measurement |
| ☐ | ☒ | A statement on whether measurements were taken from distinct samples or whether the same sample was measured repeatedly |
| ☐ | ☒ | The statistical test(s) used AND whether they are one- or two-sided<br>*Only common tests should be described solely by name; describe more complex techniques in the Methods section.* |
| ☐ | ☒ | A description of all covariates tested |
| ☐ | ☒ | A description of any assumptions or corrections, such as tests of normality and adjustment for multiple comparisons |
| ☐ | ☒ | A full description of the statistical parameters including central tendency (e.g. means) or other basic estimates (e.g. regression coefficient) AND variation (e.g. standard deviation) or associated estimates of uncertainty (e.g. confidence intervals) |
| ☐ | ☒ | For null hypothesis testing, the test statistic (e.g. $F$, $t$, $r$) with confidence intervals, effect sizes, degrees of freedom and $P$ value noted<br>*Give P values as exact values whenever suitable.* |
| ☒ | ☐ | For Bayesian analysis, information on the choice of priors and Markov chain Monte Carlo settings |
| ☒ | ☐ | For hierarchical and complex designs, identification of the appropriate level for tests and full reporting of outcomes |
| ☒ | ☐ | Estimates of effect sizes (e.g. Cohen's *d*, Pearson's *r*), indicating how they were calculated |

*Our web collection on statistics for biologists contains articles on many of the points above.*

## Software and code

Policy information about availability of computer code

| | |
|---|---|
| Data collection | Data collection software details are provided in Methods. |
| Data analysis | Data analysis software details are given in Methods. Custom script has been uploaded to our institute repository and the manuscript file has a link to it. |

For manuscripts utilizing custom algorithms or software that are central to the research but not yet described in published literature, software must be made available to editors and reviewers. We strongly encourage code deposition in a community repository (e.g. GitHub). See the Nature Portfolio guidelines for submitting code & software for further information.

## Data

Policy information about availability of data

All manuscripts must include a data availability statement. This statement should provide the following information, where applicable:

- Accession codes, unique identifiers, or web links for publicly available datasets
- A description of any restrictions on data availability
- For clinical datasets or third party data, please ensure that the statement adheres to our policy

Data availability statement is in Methods.

## Research involving human participants, their data, or biological material

Policy information about studies with human participants or human data. See also policy information about sex, gender (identity/presentation), and sexual orientation and race, ethnicity and racism.

| | |
|---|---|
| Reporting on sex and gender | Supplementary Table 1 provides details of the cells and patients (including their sex and age). |
| Reporting on race, ethnicity, or other socially relevant groupings | Values in human Pvalb neurons did not differ between males (n = 24 cells) and females (n = 12 cells) (p = 0.880, Mann-Whitney U-test). |
| Population characteristics | The human patient demographic information is defined by the participants who were recruited for and included in the study after providing informed consent. The demographic details of the human subjects are listed in Supplementary Table 1. |
| Recruitment | The participant recruitment and inclusion procedures complied with the conditions of the approved ethics licences. Written informed consent was obtained from all patients prior to surgery. For participants under 18 years of age, consent was obtained from a parent or legal guardian. |
| Ethics oversight | Human studies were approved by the Regional Human Investigation Review Board (reference 75/2014) and the National Scientific and Research Ethics Committee (ETT TUKEB; license BM/25042-1/2024). |

Note that full information on the approval of the study protocol must also be provided in the manuscript.

# Field-specific reporting

Please select the one below that is the best fit for your research. If you are not sure, read the appropriate sections before making your selection.

☒ Life sciences ☐ Behavioural & social sciences ☐ Ecological, evolutionary & environmental sciences

For a reference copy of the document with all sections, see nature.com/documents/nr-reporting-summary-flat.pdf

# Life sciences study design

All studies must disclose on these points even when the disclosure is negative.

| | |
|---|---|
| Sample size | Unless stated otherwise, sample sizes were determined based on prior electrophysiological studies in rodent neurons, with 10–30 cells per species being used. Similarly, sample sizes for molecular analyses were set at 10–30 cells per reaction, consistent with previous studies. |
| Data exclusions | The analyses were conducted without the exclusion of any data. |
| Replication | The reproducibility of the experiments was ensured through the experimental design (e.g. the use of an internal control baseline) and by employing large data sets (with at least n = 10 observations) to guarantee the statistical power of the tests and comparisons. Appropriate statistical analyses were selected to minimise random false positive errors. |
| Randomization | The data sample units (i.e. individual cells) were collected with the aim of obtaining a similar number of cells from each individual human or mouse. |
| Blinding | The experimenters were aware of the type of experimental design used. |

# Reporting for specific materials, systems and methods

We require information from authors about some types of materials, experimental systems and methods used in many studies. Here, indicate whether each material, system or method listed is relevant to your study. If you are not sure if a list item applies to your research, read the appropriate section before selecting a response.

### Materials & experimental systems

| n/a | Involved in the study | |
|---|---|---|
| ☐ | ☒ | Antibodies |
| ☒ | ☐ | Eukaryotic cell lines |
| ☒ | ☐ | Palaeontology and archaeology |
| ☐ | ☒ | Animals and other organisms |
| ☒ | ☐ | Clinical data |
| ☒ | ☐ | Dual use research of concern |
| ☒ | ☐ | Plants |

### Methods

| n/a | Involved in the study | |
|---|---|---|
| ☒ | ☐ | ChIP-seq |
| ☒ | ☐ | Flow cytometry |
| ☒ | ☐ | MRI-based neuroimaging |

## Antibodies

Antibodies used

Primary antibodies included goat anti-pv polyclonal (1:1000, PVG213, SWant, Switzerland, https://www.labome.com/product/SWant/PVG213.html); rabbit anti-Kv3.1 polyclonal (1:500, 242-0P, Synaptic Systems, https://sysy.com/product/242003#list).; rabbit Anti-GIRK1 polyclonal (1:200, APC-005, https://www.alomone.com/p/anti-kir3-1-girk1/APC-005?srsltid=AfmBOoq8Ci8aXbBSAwiOBUMrXIjVvTu5etrTOGDtb2Jw948pwyvRUuB); rabbit Anti-GIRK2 antibody monoclonal (1:200, EPR23841-83, https://www.abcam.com/en-us/products/primary-antibodies/girk2-antibody-epr23841-83-ab259909); rabbit Anti-Kir3.3 polyclonal antibody (1:200, PA5-67106, https://www.thermofisher.com/antibody/product/Kir3-3-KCNJ9-Antibody-Polyclonal/PA5-67106); rabbit AntiKir2.3 polyclonal antibody (1:200, APC-032, https://www.alomone.com/p/anti-kir2-3/APC-032?srsltid=AfmBOoqN4Qb9PE2Dr8UxTqvk49bGopwPjQwLLTM_udmQCR8bhnUyGnu4). Secondary antibodies were: DARb Alexa 647–conjugated donkey anti-rabbit (1:200, Abcam, www.abcam.com); DAGt Cy3-conjugated donkey anti-goat (1:400, Jackson ImmunoResearch www.jacksonimmuno.com); DARb Cy3-conjugated donkey anti-rabbit (1:400, Jackson ImmunoResearch www.jacksonimmuno.com); DARb Alexae 488-conjugated donkey anti-rabbit (1:400, Jackson ImmunoResearch www.jacksonimmuno.com);
DAGt Alexa 488-conjugated donkey anti-goat (1:400, Jackson ImmunoResearch www.jacksonimmuno.com).

Validation

Links to the ab validation (including original references) are given with the primary ab description above.

## Animals and other research organisms

Policy information about studies involving animals; ARRIVE guidelines recommended for reporting animal research, and Sex and Gender in Research

Laboratory animals

5–12-week-old heterozygous B6.129P2-Pvalbtm1(cre)Arbr/J mice (stock 017320, B6 PVcre line, Jackson Laboratory, Bar Harbor, ME, USA) crossed with the Ai9 reporter line, enabling tdTomato expression in Pvalb neurons.

Wild animals

Wild animals were used in the study.

Reporting on sex

Both sexes were included. There was no difference in the data for the two sexes.

Field-collected samples

Not used in the study.

Ethics oversight

All animal procedures were approved by the Governmental Office of Animal Health and Welfare (permit CS/I01/03036-2/2024) and the University of Szeged Ethics Committee.
Human studies were approved by the Regional Human Investigation Review Board (reference 75/2014) and the National Scientific and Research Ethics Committee (ETT TUKEB; license BM/25042-1/2024). All procedures conformed to the principles of the Declaration of Helsinki.

Note that full information on the approval of the study protocol must also be provided in the manuscript.

## Plants

Seed stocks

N/A

Novel plant genotypes

N/A

Authentication

N/A
