# Supplementary material for: Conserved Kir channel mechanisms governing intrinsic excitability in human and rodent parvalbumin neurons
Source: Commun Biol. 2026 Apr 13;9:806. doi: 10.1038/s42003-026-10063-9 (PMC13265741; doi:10.1038/s42003-026-10063-9)
Supplement: Supplementary file 1 — Transparent Peer Review file [file 42003_2026_10063_MOESM1_ESM.pdf]

nature portfolio

Peer Review File

Conserved Kir channel mechanisms governing intrinsic excitability in human and rodent parvalbumin neurons

Corresponding Author: Dr Karri Lamsa

This file contains all reviewer reports in order by version, followed by all author rebuttals in order by version.

Version 0:

Reviewer comments:

Reviewer #1

(Remarks to the Author)
Summary: Identifying shared and divergent principles of neuronal physiology between humans and rodents is very relevant to the field of physiology and neuroscience. Besides well-established differences in HCN channels, the author have focused on Kir channels in parvalbumin neurons. The authors performed experiments over a wide range of methodology at high quality in rarely accessible and valuable human cortical tissue. In the main electrophysiology experiments, they found Kir-dependent conductance at hyperpolarized potential but no species-difference. They further verified the transcriptomic expression and membrane localization of Kir channels in both species. The only cross-species difference was observed in the overall input resistance which the authors modelled to predict a stronger Kir conductance in mouse PV neurons. Overall, the study addresses a specific but nonetheless important topic with high quality experiments in rare human tissue. While they have not found any Kir-specific differences across the species, these empirical results in human PV interneurons represent an important contribution to the field of comparative neuron physiology.

Comments:
1) The empirical results on synaptic inhibition (Fig6) are somewhat limited in novelty and significance. The authors show hyperpolarization of the membrane potential by IPSPs elicited by NGF neurons, similar to Olah et al. 2007. Considering this and the fact that they did not pharmacologically isolate GABA-B-mediated Kir conductances in the paired recordings, the abstract claiming “demonstration of physiological Kir activation” could be attenuated. While the authors simulated how IPSPs could shunt incoming EPSPs, they have not been empirically verified. If the authors wish to demonstrate a more physiological example of how Kir channel activation affects input excitability, they might consider eliciting EPSP from paired pyramidal neurons while clamping the PV neurons at different RMPs while blocking HCN channels.

2) Fig1C: show line to indicate mean/mean (depending on parametric/non-parametric statistical test performed).

3) The simulation result follows basic membrane physiology principles. Nonetheless, it helps to quantify the implications of the empirically shown differences in overall membrane input resistance. This point could be made clearer in the manuscript and the figure, e.g. visualizing the parameter space that matches the empirical Vm and Rin/Gleak in both species.

4) Ending the discussion with a paragraph on opioid receptors without any prior motivation seems a bit abrupt. May it would be more useful for the reader to add a broader sentence to conclude the significance of the results with respect to what has been discussed.

Reviewer #2

(Remarks to the Author)
Summary of the Work
This manuscript presents a comprehensive study on the role of inwardly rectifying potassium (Kir) channels in regulating the input excitability of parvalbumin-expressing (Pvalb) interneurons in both human and mouse neocortex. Employing a multidisciplinary strategy including whole-cell patch-clamp recordings, single-cell transcriptomics (patch-seq), immunohistochemistry, super-resolution microscopy (STORM), and computational modeling, the authors uncover a conserved voltage-dependent decrease in input resistance (Rin) during hyperpolarization mediated primarily by Kir3.1 and

Kir3.2 channels. Functional modeling reveals that Kir-mediated shunting inhibition, particularly during synaptic input from neurogliaform (NGF) cells, plays a key role in dampening excitatory signals. While the study emphasizes species conservation, it also points to unique expression profiles and inhibitory dynamics between humans and mice.

This study has the following scientific merits and strengths: First, the study uses both human surgical tissue and mouse models to address evolutionary conservation, a notable strength rarely seen in neuroscience research. Secondly, the combination of electrophysiology, patch-seq, immunohistochemistry, pharmacology (Ba^{2+} blockade), and computational modeling provides a multi-layered view of Kir function. Thirdly, the authors demonstrate how Kir channels, particularly Kir3.1 and Kir3.2, modulate Rin and mediate shunting inhibition via NGF cell input, contributing to inhibitory control in cortical microcircuits. Finally, the modeling approach effectively simulates the biophysical consequences of Kir activation in human PV neurons and provides testable predictions regarding Gleak and GKir relationships.

I have some minor concerns:
1. The rationale for selecting NGF→PV synapses for shunting analysis is not well justified, and alternative sources of inhibition are not considered.
2. Figure 1b panels are presented in a non-intuitive order (b2/b4 logically precede b1/b3).
3. Page 5: "If PVALB expression was undetectable, then the mRNAs of genes indicative of a neuron type with extremely low Pvalb expression in humans was identified…" The grammatical structure should be corrected to ensure subject-verb agreement, i.e., "were identified."
4. Please clarify whether Rin measurements in Figure 6 were obtained through synaptic or current-step protocols.
5. The abbreviation for neurogliaform cells (NGF) should be standardized throughout the text.
6. The suggestion that PV neuron subtype diversity contributes to interspecies differences in Kir activity should be elaborated. Is this molecular, morphological, or electrophysiological diversity? Please cite some literatures.

Reviewer #3

(Remarks to the Author)
The manuscript by Furdan and coauthors compares the regulation of excitability by Kir channels in cortical parvalbumin interneurons in human vs mouse slices. The authors find similar results, which suggest the evolutionary conservation of this archetypal mechanism.
Although many of the authors have made seminal contributions to the field and are highly respected, I find this particular work underwhelming and maybe more appropriate for a different journal for the following reasons.

Major Points

1) Despite the high interest and importance of studying the properties of human tissue, results are mostly negative, with no differences found between human and mouse tissue. Although this remains an interesting conclusion, the work is, in my opinion, more appropriate for a different journal.

2) The style is often too technical and there is little effort to reach a general audience. For example, the authors measure and illustrate the input rectification ratio and find that there is a different association with the initial Rin in human vs mouse cells (Fig 1d). However, this finding is neither explained nor discussed. A similar problem is the lack of correlation between the rectification index and KCNJ mRNA expression (Fig 3c). Wouldn't a correlation be expected here?

3) The way cells are classified is rather inconsistent. In human tissue, some cells were identified by direct Pvalb immunolabeling, others by patch seq and others (with undetectable PVALB expression) by the Allen Institute Neuron Type Classification system. In mouse tissue, tdTomato was used as a proxy for Pvalb. Furthermore, only a single reconstruction from human tissue is included. It would have been interesting to see the variability of the sample.

4) The electrophysiological data illustrated in Fig 1 were collected in the presence of ZD7288. Why? Ih is physiologically present in parvalbumin interneurons, and both its activation and its deactivation will affect the measurement of Rin. For example, suppose that Ih was expressed at highly different densities in human vs mouse neurons, wouldn't this change interpretations and conclusions? In addition, ZD7288 at the concentration used in the paper has been reported to have off-target effects on other voltage-gated channels (Sanchez-Alonso et al., Neurosci Lett, 2008, Wu et al., Channels, 2012). This general point is even strengthened by the inconsistency with the modeling simulations of the last two figures, which include the H conductance in the postsynaptic cell.

5) The data of Fig 4 are not easy to understand. First, the terminology used is confusing. There is no such thing as an "extracellular membrane". The authors most likely refer to the "plasma membrane", which has an "extracellular side", but I do not think the authors are able to identify this here. In fact, it is also difficult to understand how the zero point in the abscissa of panels a2, b2, c2 and d2 was defined and/or identified. Second, the plots in a2, b2, c2 and d2 are difficult to interpret. It would seem that the peak of fluorescence for Kir channels is on the intracellular side of the plasma membrane, if it is not even completely intracellular. Why? Were the primary antibodies used mono- or polyclonal? Is the localization of the epitope targeted by the antibodies used known? Third, why does the Kir 3.2 immunofluorescence in Fig 4d2 extend at peak levels for several microns on the extracellular side? Fourth, Pvalb and TdTomato are assumed to have identical intracellular distributions. As they are both soluble proteins, this is quite possible, but a caveat sentence indicating that these are different proteins should be added. Fifth and last, the claim that "the STORM immunofluorescence images highlight the localization of individual fluorophore molecule signals at the extracellular membrane" is rather overstated. Apart from the use of

“extracellular membrane” mentioned before, this is, in my opinion at least, a very subjective interpretation, given the high background level, and the exclusively qualitative analysis based on single selected images.

6) The data on synaptic inhibition (Fig 6) are obtained in a few pairs from human tissue and there is no comparison to mouse data.

Minor Points

1) Throughout the text, it is difficult to understand how many cells/subjects were recorded or analyzed and what statistical tests were used.

2) Some statements are not consistent with the illustrations. For example (Figs 2b1, b2): “The progressive reduction of Rin and its blockade by micromolar Ba2+ levels”. The reduction of Rin is not “blocked” by Ba2+ because it is still present both in Fig2a1 and 2b1. If anything, the slope of the plots is only slightly reduced. This should be analyzed quantitatively.

3) Some panels and fonts of the illustrations are too small to be readable in printed form. For example, Figs 5c1, c2 and Fig 6b.

Version 1:

Reviewer comments:

Reviewer #1

(Remarks to the Author)
The authors have substantially revised the manuscript by including additional data and new approaches to convincingly support their claim. By focusing the key conclusion on conserved Kir channel mechanisms to compensate for species-specific differences in intrinsic excitability, the authors made the conceptual advance and novelty more clear. The manuscript has been greatly improved and will be of high relevance for the community and the broader audience.

Reviewer #2

(Remarks to the Author)
The authors have adequately addressed my concerns, and the revised manuscript has been substantially improved.

Reviewer #3

(Remarks to the Author)
Unfortunately, I do not believe that the concerns raised in my previous review have been adequately addressed.

1) Although the study is of interest, particularly due to the use of human tissue, the results remain relatively limited in scope and impact. In my view, the work would be more suitable for a specialized journal.

2) I remain concerned that the use of different criteria for identifying parvalbumin-positive cells reduces the rigor of the study. If extended electrophysiological recordings precluded single-cell gene expression profiling, parvalbumin immunostaining could have been applied consistently across all cells. Furthermore, comparing native parvalbumin expression in human neurons with tdTomato fluorescence driven by the parvalbumin promoter in mouse neurons remains suggestive rather than conclusive.

3) I maintain that measuring input resistance (Figure 1) in the presence of ZD7288 creates a highly artificial and non-physiological condition, as detailed in my original review. While I understand the rationale for using ZD7288 in separate experiments to isolate Kir channel effects during Ba^{2+} application, this does not justify its use in the experiments shown in Figure 1. An alternative approach would have been to record baseline properties without ZD7288 (for Figure 1) and then apply it prior to Ba^{2+} (For Figure 2).

4) The section on mRNA expression of Kir channel subunits remains weak. As the authors acknowledge, mRNA levels may not reflect protein expression, and the conclusion that human and mouse parvalbumin neurons express multiple Kir channel types is modest. The functional significance of this diversity is not discussed.

5) The computational modeling and dynamic clamp experiments were conducted with HCN channels absent or blocked, again representing a non-physiological condition. Although consistency across experiments is desirable, this should have been achieved by performing the measurements in Figure 1 and the modeling/dynamic clamp experiments in Figure 7 under control conditions (with intact HCN conductance) rather than under HCN blockade.

Open Access This Peer Review File is licensed under a Creative Commons Attribution 4.0 International License, which permits use, sharing, adaptation, distribution and reproduction in any medium or format, as long as you give appropriate credit to the original author(s) and the source, provide a link to the Creative Commons license, and indicate if changes were made.
In cases where reviewers are anonymous, credit should be given to 'Anonymous Referee' and the source.
The images or other third party material in this Peer Review File are included in the article’s Creative Commons license, unless indicated otherwise in a credit line to the material. If material is not included in the article’s Creative Commons license and your intended use is not permitted by statutory regulation or exceeds the permitted use, you will need to obtain permission directly from the copyright holder.
To view a copy of this license, visit https://creativecommons.org/licenses/by/4.0/

We thank the Editor and the three expert Reviewers for their constructive and thoughtful evaluation of our previous manuscript. We have substantially revised the study and the manuscript in response to their comments. The revised version has been extensively rewritten and now includes new experimental data, new analyses, and new figures, as detailed below. We believe these changes have significantly strengthened the rigor, clarity, and impact of the work. Below, we provide a point-by-point response to the Editorial and Reviewer comments.

Responses to Editorial Comments:

In particular, additional explanations are vital, for example,

-why you chose "NGF → PV synapses for shunting analysis" as pointed out by Reviewer #2,

Our reply: We now explain more clearly in the manuscript that we specifically focused on NGF cell–evoked IPSPs in Pvalb neurons because these synaptic connections generate well-characterized slow IPSPs with defined kinetics in both human and rodent fast-spiking postsynaptic neurons. These IPSPs are mediated by GABA-B receptor–dependent activation of Kir channels. In addition, we have added new computational simulations and dynamic-clamp experiments to directly demonstrate the IPSP-mediated shunting inhibition effect in human and mouse Pvalb neurons.

-or why cells were classified in different ways (see Reviewer #3, PV immunolabeling, patch seq, etc.). Especially, the last point seems important as a proper classification of the cells you recorded seems crucial.

The majority of neurons were classified based on their gene expression profiles using the Allen Institute cell-type identity classification system, which is openly available. However, successful harvesting of nuclei and mRNA after prolonged whole-cell recordings has been technically challenging in our hands; in our experience, reliable nucleus retrieval and mRNA analysis are typically achieved when the whole-cell recording duration does not exceed ~15 minutes.

For experiments requiring extended recording times, such as Kir channel blocker wash-in protocols, slices were fixed after recording and neuronal identity was determined by post hoc immunofluorescence. Cells subjected to Pvalb immunoreactivity included neurons exposed to $Ba^{2}+$ as well as cells studied using dynamic-clamp protocols. In these experiments, cell identity was therefore established by Pvalb immunolabeling rather than patch-sequencing.

-I would also be interested in the firing patterns observed in PV+ neurons, in particular whether fast-spiking was observed.

Our reply: The action potential firing pattern was not systematically studied in cells in this study of Kir channels (which are activated by hyperpolarisation). Firing pattern analysis involves ion channels that are activated or inactivated by depolarisation at resting Vm, which is a different area of study. Therefore, analysis of the firing pattern was omitted from this work.

-In addition to these points, there were a few other suggestions. Reviewer #1, for example, pointed out that you had "simulated how IPSPs could shunt incoming EPSPs", but that you had not "empirically verified" this. For further details, please see the comments below.
Our reply: We now present additional experiments using dynamic clamp to demonstrate EPSP shunting in hyperpolarising synaptic potentials in real human and mouse cells. These experiments align with updated and more comprehensive simulations from the computational model.

-we ask that your manuscript complies with our editorial policies.
Our reply: Checked. We for instance added individual data points alongside boxplots to show data distribution in graphs.

-It's mandatory to provide access to the numerical source data for graphs and charts either through a repository or by providing the data in a Supplementary Data file (in excel format).
Our reply: The data source files for the results were uploaded as worksheet files. In addition, custom scripts with instructions on how to install and use them to analyse the source data were uploaded to our institute's repository site, and a link to this site was provided in the manuscript text.

-All blots/gels must be accompanied by size markers in every figure panel. Uncropped and unedited blot/gel images must be included as Supplementary Figure(s) in the Supplementary Information pdf.
Our reply: All anatomical data images include a scale bar.

-Please ensure that you have complied with the data deposition policies at the Nature Portfolio. Please ensure that you have complied with our policies on research involving animals and humans.
Our reply: The details of ethical licences and licence ID codes are given in the specified paragraph in the 'Methods' section.

-Please follow the ARRIVE guidelines for reporting animal experiments. Please fully complete an ARRIVE checklist including both the essential and recommended set of items (adding information to the manuscript where needed) and upload this with your revised manuscript.
Our reply: The checklist has been completed and uploaded with the manuscript files.

-Please also see our revision checklist for guidance on formatting the manuscript and complying with our policies. A comprehensive guide to our formatting requirements for final submissions is also available for your reference here.
Our reply: The checklists are completed.

Our brief summary of the changes to the figures and tables is as follows:
-Fig. 1c1-2 and 1D have been updated with new data. Data are now shown as box plots and individual values.
-Fig. 2: layout change. In addition, Fig. 2C has been updated with new data, and the old Fig. C2 has been removed and replaced with a new table (Table 1), which demonstrates

the effect of Ba^{2+} and uses statistical analysis to show that the effect of Ba^{2+} is similar in both species.
-Fig. 3 has a modified layout for clarity (symbols and labelling).
-Fig. 4 is a new figure with new experiments presenting the results of a systematic study analysing the subcellular localisation of the immunofluorescence signal for the four Kir channel types expressed in both human and mouse Pvalb neurons.
-Fig. 5 is a new figure with new data analysing the subcellular localisation of the immunofluorescence signal of the four Kir channel types using signal area segmentation (with our custom made script) in confocal microscope images.
Figures 4 and 5 show systematic analyses of Kir channel cellular localisation based on confocal immunofluorescence microscopy. These systematic large volume analyses now replace the STORM superresolution images of individual cells presented in the previous version of the manuscript. The conclusions in the revised manuscript are based on large-scale microcircuit analyses using these methods.
-Fig. 6: Minor changes to the layout of d1 have been made for clarity.
-Fig. 7: Fig. 7a1-3 has a new organisation and presentation to improve clarity. Figures 7b1-2 show new simulation experiments under different Kir activation conditions in a model cell. Figures 7c1-2 show new experiments with dynamic clamp in human Pvalb neurons, demonstrating shunting inhibition of EPSPs by Kir channels.

Reviewer #1 (Remarks to the Author):

Summary: Identifying shared and divergent principles of neuronal physiology between humans and rodents is very relevant to the field of physiology and neuroscience. Besides well-established differences in HCN channels, the author have focused on Kir channels in parvalbumin neurons. The authors performed experiments over a wide range of methodology at high quality in rarely accessible and valuable human cortical tissue. In the main electrophysiology experiments, they found Kir-dependent conductance at hyperpolarized potential but no species-difference. They further verified the transcriptomic expression and membrane localization of Kir channels in both species. The only cross-species difference was observed in the overall input resistance which the authors modelled to predict a stronger Kir conductance in mouse PV neurons.
Overall, the study addresses a specific but nonetheless important topic with high quality experiments in rare human tissue. While they have not found any Kir-specific differences across the species, these empirical results in human PV interneurons represent an important contribution to the field of comparative neuron physiology.

1) The empirical results on synaptic inhibition (Fig6) are somewhat limited in novelty and significance.
Our reply: We have added new empirical data using dynamic clamp to the figure (currently Fig. 7) to demonstrate how Kir activity inhibits EPSPs in both human and mouse Pvalb neurons, whether it is non-synaptic or synaptic.
The authors show hyperpolarization of the membrane potential by IPSPs elicited by NGF neurons, similar to Olah et al. 2007. Considering this and the fact that they did not

pharmacologically isolate GABA-B-mediated Kir conductances in the paired recordings, the abstract claiming "demonstration of physiological Kir activation" could be attenuated.
Our reply: We rewrote the entire manuscript, including the abstract.

-While the authors simulated how IPSPs could shunt incoming EPSPs, they have not been empirically verified. If the authors wish to demonstrate a more physiological example of how Kir channel activation affects input excitability, they might consider eliciting EPSP from paired pyramidal neurons while clamping the PV neurons at different RMPs while blocking HCN channels.
Our reply: We have added recordings of human and mouse Pvalb neurons using dynamic clamp to demonstrate EPSP shunting inhibition by Kir-mediated IPSPs (HCN-channels blocked). These new experiments are illustrated in Fig. 7c1-2.

2) Fig1C: show line to indicate mean/mean (depending on parametric/non-parametric statistical test performed).
Our reply: We have added box plots showing median and upper and lower quartiles to Figures 1C1 and 1C2.

3) The simulation result follows basic membrane physiology principles. Nonetheless, it helps to quantify the implications of the empirically shown differences in overall membrane input resistance. This point could be made clearer in the manuscript and the figure, e.g. visualizing the parameter space that matches the empirical Vm and Rin/Gleak in both species.
Our reply: We have provided a more comprehensive illustration of the Vm steps in the updated Figures 2A2 and 2B2, which show the Vm steady state in the traces. We also provide a clearer explanation of the measurement of Rin and rRin (see Methods: 'Input resistance (Rin) was calculated as ΔVm/ΔI from the steady-state membrane potentials reached during voltage steps in the presence of ZD7288 (50 µM).'

4) Ending the discussion with a paragraph on opioid receptors without any prior motivation seems a bit abrupt. May it would be more useful for the reader to add a broader sentence to conclude the significance of the results with respect to what has been discussed.
Our reply: We have completely rewritten the discussion section. We shortened the sections on opioid receptors and Kir channels, and added a concluding paragraph.

Reviewer #2 (Remarks to the Author):

Summary of the Work
This manuscript presents a comprehensive study on the role of inwardly rectifying potassium (Kir) channels in regulating the input excitability of parvalbumin-expressing (Pvalb) interneurons in both human and mouse neocortex. Employing a multidisciplinary strategy including whole-cell patch-clamp recordings, single-cell transcriptomics (patch-seq), immunohistochemistry, super-resolution microscopy (STORM), and computational modeling, the authors uncover a conserved voltage-dependent decrease in input resistance (Rin) during hyperpolarization mediated primarily by Kir3.1 and Kir3.2 channels. Functional

modeling reveals that Kir-mediated shunting inhibition, particularly during synaptic input from neurogliaform (NGF) cells, plays a key role in dampening excitatory signals. While the study emphasizes species conservation, it also points to unique expression profiles and inhibitory dynamics between humans and mice.

This study has the following scientific merits and strengths: First, the study uses both human surgical tissue and mouse models to address evolutionary conservation, a notable strength rarely seen in neuroscience research. Secondly, the combination of electrophysiology, patch-seq, immunohistochemistry, pharmacology (Ba^{2+} blockade), and computational modeling provides a multi-layered view of Kir function. Thirdly, the authors demonstrate how Kir channels, particularly Kir3.1 and Kir3.2, modulate Rin and mediate shunting inhibition via NGF cell input, contributing to inhibitory control in cortical microcircuits. Finally, the modeling approach effectively simulates the biophysical consequences of Kir activation in human PV neurons and provides testable predictions regarding Gleak and GKir relationships.

I have some minor concerns:
1. The rationale for selecting NGF→PV synapses for shunting analysis is not well justified, and alternative sources of inhibition are not considered.
Our reply: We have rewritten the results, discussion and figure legends. In the revised Figure 7, we provide a clearer explanation of why we selected the NGF-PV synapse for the Kir channel study. We also added new simulations and experiments using dynamic clamp to demonstrate the role of Kir channels in the inhibition of EPSPs in Pvalb neurons. These experiments analyse the non-synaptic and synaptic Kir channels (which underlie the slow IPSP) in EPSP inhibition, demonstrating how these two Kir components operate both independently and together to suppress somatic EPSPs in human and mouse Pvalb neurons.

2. Figure 1b panels are presented in a non-intuitive order (b2/b4 logically precede b1/b3).
Our response: We have changed the layout of Figure 2 so that the data is presented in a more logical way.

3. Page 5: "If PVALB expression was undetectable, then the mRNAs of genes indicative of a neuron type with extremely low Pvalb expression in humans was identified..." The grammatical structure should be corrected to ensure subject-verb agreement, i.e., "were identified."
Our reply: This is now changed as *"...neurons in which PVALB transcripts were undetectable, cell identity was assigned using the Allen Institute Neocortical Neuron Type Classification System (https://knowledge.brain-map.org/mapmycells/process/), which classifies layer 2/3 neocortical neuron types based on their global gene expression profiles".*

4. Please clarify whether Rin measurements in Figure 6 were obtained through synaptic or current-step protocols.
Our reply: This has now been clarified in the legend of the figure (currently Fig. 7): '*...as measured using current-step protocols in the absence of IPSPs.*'

5. The abbreviation for neurogliaform cells (NGF) should be standardized throughout the text.

Our reply: Neurogliaform cells are consistently called now as 'neurogliaforms cells' or 'NGFs' throughout the manuscript.

6. The suggestion that PV neuron subtype diversity contributes to interspecies differences in Kir activity should be elaborated. Is this molecular, morphological, or electrophysiological diversity? Please cite some literatures.

Our reply: Although there is no direct evidence in the literature to show this, we proposed the idea based on Allen Institute gene expression analyses. We added a citation to the original Allen Institute publication describing interneuron types and subtypes in the neocortex by gene expression, to illustrate the variability of mRNA profiles between cell subtypes.

We moderated this statement in results as follows:
"This species difference may reflect the greater variability and wider range of baseline input resistance in human compared with mouse cells[38], potentially arising from increased diversity of Pvalb interneuron subtypes in the human neocortex [1, 39]".

We formulated this in Discussion as follows: *"Although Kir-mediated inhibition was robust in both species, we observed species-specific variability. In humans, Pvalb neurons with either high or low Rin could exhibit strong or weak Kir-mediated inhibition, whereas in mice the magnitude of Kir inhibition correlated with Rin. This difference may reflect greater genetic and transcriptomic diversity among Pvalb subtypes in the human compared with the mouse neocortex. Consistent with this interpretation, Kir gene expression varies across Pvalb neuron types between species [7, 39]"*

Reviewer #3 (Remarks to the Author):

The manuscript by Furdan and coauthors compares the regulation of excitability by Kir channels in cortical parvalbumin interneurons in human vs mouse slices. The authors find similar results, which suggest the evolutionary conservation of this archetypal mechanism. Although many of the authors have made seminal contributions to the field and are highly respected, I find this particular work underwhelming and maybe more appropriate for a different journal for the following reasons.

Major Points

1) Despite the high interest and importance of studying the properties of human tissue, results are mostly negative, with no differences found between human and mouse tissue. Although this remains an interesting conclusion, the work is, in my opinion, more appropriate for a different journal.

Our reply: We thank the reviewer for these insightful comments. In the revised manuscript, we emphasize that homologous Pvalb neurons in humans have evolved toward a

conserved, archetypal excitability phenotype, despite substantial differences in baseline excitability between species. Using simulations (current Fig. 6), we demonstrate a mechanistic adaptation toward this archetypal electrical phenotype: human Pvalb neurons reach comparable functional behavior with markedly lower Kir conductance than mouse neurons.

We also mentioned in the manuscript that the convergence of Kir-mediated inhibition in human and rodent neurons is particularly noteworthy given that other regulators of excitability—such as HCN1/HCN2 and Kv1.1/Kv1.2 channels—differ significantly between species (Szegedi et al., 2023; Bakos et al., 2025; both cited in the manuscript). We formulate this in the 'Discussion' as follows: "*This principle contrasts with other conductances, such as HCN or Kv1 channels, whose density and localization differ in Pvalb neurons markedly between species, highlighting Kir channels as a conserved regulator of interneuron excitability* [15, 16, 38]."

We have also expanded our subcellular localization analysis to include all four Kir channel types expressed in both species (identified at the mRNA level by patch-sequencing). These analyses reveal a species-specific difference: human Pvalb neurons exhibit low abundance and rare somatic membrane localization of Kir3.2, whereas in mouse Pvalb neurons all four Kir channel types are abundantly present at the soma. To robustly support this conclusion, we developed two parallel and independent approaches, presented in new Figures 4 and 5. Figure 5 shows Kir-channel localization based on area segmentation of immunofluorescence signals from confocal z-stacks, implemented using a custom analysis script developed for this study.

Finally, we added new dynamic clamp experiments to experimentally test the predictions of the simulations (which were also re-run), directly demonstrating Kir-mediated shunting inhibition in recorded neurons.

2) The style is often too technical and there is little effort to reach a general audience.
Our reply: We have rewritten the manuscript introduction and discussion, as well as large parts of the results section and figure legends, to make the manuscript clearer and more readable for a lay audience.

-For example, the authors measure and illustrate the input rectification ratio and find that there is a different association with the initial Rin in human vs mouse cells (Fig 1d). However, this finding is neither explained nor discussed.
Our reply: We now provide a possible explanation for this finding in the revised manuscript, supported by appropriate references. In Results we mention: "*This species difference may reflect the greater variability and wider range of baseline input resistance in human compared with mouse cells*[38]*, potentially arising from increased diversity of Pvalb interneuron subtypes in the human neocortex* [1, 39]" In Discussion we write: "*Although Kir-mediated inhibition was robust in both species, we observed species-specific variability. In humans, Pvalb neurons with either high or low Rin could exhibit strong or weak Kir-mediated inhibition, whereas in mice the magnitude of Kir inhibition correlated with Rin. This*

difference may reflect greater genetic and transcriptomic diversity among Pvalb subtypes in the human compared with the mouse neocortex. Consistent with this interpretation, Kir gene expression varies across Pvalb neuron types between species [7, 39]

-A similar problem is the lack of correlation between the rectification index and KCNJ mRNA expression (Fig 3c). Wouldn't a correlation be expected here?
Our reply: We explain this now in the manuscript (with references): "*However, cytoplasmic KCNJ mRNA levels do not necessarily predict the abundance or subcellular localization of Kir proteins in the somatic membrane, as transcript levels captured at the time of nuclear collection may poorly reflect cellular protein abundance, likely due to burst-like rather than steady-state gene expression.*"

3) The way cells are classified is rather inconsistent. In human tissue, some cells were identified by direct Pvalb immunolabeling, others by patch seq and others (with undetectable PVALB expression) by the Allen Institute Neuron Type Classification system. In mouse tissue, tdTomato was used as a proxy for Pvalb.
Our reply: The majority of neurons were classified by gene expression profile using the Allen Institute's openly available cell-type identity classification system. However, we have had less success harvesting mRNA and nuclei from cells after longer electrophysiological whole-cell recordings. In experiments requiring a Kir channel blocker wash-in (with a baseline and barium wash-in), we fixed the slice and tested cell identity using immunofluorescence. Cells tested with an immunoreaction for Pvalb included those exposed to barium, as well as neurons studied with dynamic clamp, for example.

For the mouse, we utilised an established method of using genetically labelled cells (Bakos et al., 2025; cited in this study). We also tested ten of these cells with patch-sequencing for PVALB gene expression (Fig. 1a4).

The rationale behind the variable identification method is explained in the Results section: "*Eighteen neurons were confirmed as Pvalb-positive by immunostaining after intracellular biocytin filling. Twenty-one cells were identified using patch-seq. Eleven cells exhibited detectable levels of PVALB mRNA. For the remaining ten patch-sequenced neurons in which PVALB transcripts were undetectable, cell identity was assigned using the Allen Institute Neocortical Neuron Type Classification System (https://knowledge.brain-map.org/mapmycells/process/), which classifies layer 2/3 neocortical neuron types based on their global gene expression profiles*".

-Furthermore, only a single reconstruction from human tissue is included. It would have been interesting to see the variability of the sample.
Our reply: The reconstructions in the manuscript serve to demonstrate how cell identity was confirmed (for immunohistochemistry to visualise axon of the recorded neuron). Regarding the narrative of the research manuscript, we found that presenting comprehensive individal cell reconstructions or various reconstructed cells is outside the scope of the present story since this manuscript is not aiming for anatomical microstructure (axon, dendrite) analyses.

4) The electrophysiological data illustrated in Fig 1 were collected in the presence of ZD7288. Why? Ih is physiologically present in parvalbumin interneurons, and both its activation and its deactivation will affect the measurement of Rin. For example, suppose that Ih was expressed at highly different densities in human vs mouse neurons, wouldn't this change interpretations and conclusions? In addition, ZD7288 at the concentration used in the paper has been reported to have off-target effects on other voltage-gated channels (Sanchez-Alonso et al., Neurosci Lett, 2008, Wu et al., Channels, 2012).

Our reply: HCN channels were systematically blocked to isolate the effect of Kir channels on input resistance during Ba^{2+} application. This is now explicitly stated in the Results: "*All experiments were conducted in the presence of the HCN channel blocker ZD7288 (50 µM) to isolate Kir channel–mediated effects on Rin across hyperpolarized and depolarized Vm values.*"

The concentration of ZD7288 used is consistent with that of commonly reported in the literature. Under these conditions, Ba^{2+} produced a robust and significant effect on the input resistance of Pvalb neurons at hyperpolarized membrane potentials in both species.

-This general point is even strengthened by the inconsistency with the modeling simulations of the last two figures, which include the H conductance in the postsynaptic cell.

Our reply: We have now run the actual simulations in Fig. 7 without HCN channels. In Figure 6, we demonstrated how the simulated cell's electrical behaviour matches that of a real cell by showing that very small active HCN conductance was present in the cell. The HCN channel has been removed from the simulation in Figure 7, as well as from the new set of experiments with dynamic clamp (Figure 7), in which we demonstrated the Kir-mediated shunting effect of EPSP.

5) The data of Fig 4 are not easy to understand. First, the terminology used is confusing. There is no such thing as an "extracellular membrane". The authors most likely refer to the "plasma membrane", which has an "extracellular side",...

Our reply: We have rewritten the results section and figure legends to include all data from immunohistochemical analyses. The new data are shown in Figures 4 and 5 alongside the new text. We do not use the term 'plasma membrane' in the manuscript, but consistently refer to it as 'extracellular membrane'.

-but I do not think the authors are able to identify this here. In fact, it is also difficult to understand how the zero point in the abscissa of panels a2, b2, c2 and d2 was defined and/or identified.

Our reply: We now report the immunofluorescence intensity line analysis in more detail (see new Fig. 4), including the 0-point defined by the abrupt appearance of the intracellular marker fluorescence signal along the line when moving from the extracellular space towards the centre of the cell soma. In addition, we verified the 0-point by conducting a systematic analysis of Kv3.1 channel immunofluorescence in both species. This channel is strongly localised in the extracellular membrane, and its fluorescence profile verifies our

method of estimating the localisation of the extracellular membrane for systematic analysis. A similar approach and analysis was recently used in our studies (Szegedi et al. 2023, https://doi.org/10.1371/journal.pbio.3002001; Bakos et al. 2025, https://doi.org/10.1371/journal.pbio.3003549).

-Second, the plots in a2, b2, c2 and d2 are difficult to interpret. It would seem that the peak of fluorescence for Kir channels is on the intracellular side of the plasma membrane, if it is not even completely intracellular. Why? Were the primary antibodies used mono- or polyclonal? Is the localization of the epitope targeted by the antibodies used known?

Our reply: To answer this question, we ran a systematic analysis of various neurons based on high-resolution confocal microscope images and image stacks, using two different analysis approaches (see Figures 4 and 5). This enabled us to show the median (and quartiles) of various neurons, as well as making a statistical comparison of the immunofluorescence signal strength between defined subcellular loci. These new analyses provide statistical evidence for the localisation of channels in the membrane and cytoplasm.

-Third, why does the Kir 3.2 immunofluorescence in Fig 4d2 extend at peak levels for several microns on the extracellular side?

Our reply: Following systematic analysis of multiple cells from both species, using two independent and complementary quantitative approaches, we found that robust Kir3.2 immunoreactivity at the somatic membrane is rare in human Pvalb neurons, whereas it is readily detectable in mouse neurons. Across the human dataset, neither analysis revealed significant Kir3.2 enrichment in Pvalb neurons, despite the presence of specific immunoreactivity in the extracellular background surrounding Pvalb neuron somata.

The antibody used against Kir3.2 is monoclonal, and, as the reviewer suggests, the observed fluorescence intensity distribution may be influenced by the epitope being accessible predominantly from one side of the membrane. In contrast, the antibodies used for other Kir subtypes are polyclonal and may bind to both extracellular and intracellular epitopes. Full details of all antibodies are now provided in the Methods section, including links to the manufacturers' or distributors' documentation describing their specificity in human and mouse tissue.

-Fourth, Pvalb and TdTomato are assumed to have identical intracellular distributions. As they are both soluble proteins, this is quite possible, but a caveat sentence indicating that these are different proteins should be added.

Our reply: We have now mentioned this in the 'Results' section, along with references to other studies using similar genetically modified mice. Our formulation in the Results section is as follows: "*...mouse Pvalb neurons were examined in PVALB-tdTomato reporter mice, where tdTomato fluorescence labels PVALB-expressing cells*".

-Fifth and last, the claim that "the STORM immunofluorescence images highlight the localization of individual fluorophore molecule signals at the extracellular membrane" is rather overstated. Apart from the use of "extracellular membrane" mentioned before, this

is, in my opinion at least, a very subjective interpretation, given the high background level, and the exclusively qualitative analysis based on single selected images.
Our reply: We replaced the STORM analysis in the manuscript with two systematic confocal microscope analyses of numerous cells from both species, in order to enable statistical testing.

6) The data on synaptic inhibition (Fig 6) are obtained in a few pairs from human tissue and there is no comparison to mouse data.
We conducted experiments using dynamic clamp in both species to demonstrate the inhibitory strength of the Kir channels.

Minor Points

1) Throughout the text, it is difficult to understand how many cells/subjects were recorded or analyzed and what statistical tests were used.
Our reply: We have rewritten the manuscript and now report n-numbers, significance values and the name of statistical tests systematically in the 'Results' section and in the figure legends.

2) Some statements are not consistent with the illustrations. For example (Figs 2b1, b2): "The progressive reduction of Rin and its blockade by micromolar Ba2+ levels". The reduction of Rin is not "blocked" by Ba2+ because it is still present both in Fig2a1 and 2b1. If anything, the slope of the plots is only slightly reduced. This should be analyzed quantitatively.
Our reply: We have rewritten those parts in the manuscript. In additon, we now provide a new Table 1 with appropriate statistical analyses showing the effect of Ba^{2+} within each species and comparing the effect between species.
Table 1 legend: *"Ba^{2+} similarly abolishes voltage-dependent input resistance rectification in human and mouse Pvalb neurons. The Kir channel blocker Ba^{2+} (100 µM) significantly reduces the hyperpolarization-induced decrease in input resistance (Rin) in both human and mouse Pvalb neurons. Rectification ratios (rRin), expressed as median and interquartile range (Q1–Q3), were calculated as Rin at −90 mV relative to −70 mV under control conditions and after Ba^{2+} application. Mixed-design ANOVA revealed significant main effect of time (baseline vs. Ba^{2+}, human and mouse data pooled) indicating a consistent reduction in Rin rectification following Kir channel blockade across species. The species × time interaction p-value ($p > 0.05$) shows that the magnitude of the Ba^{2+} effect is similar between human and mouse neurons. Post hoc comparisons were performed using Wilcoxon signed-rank tests for within-species comparisons and Mann–Whitney U tests for between-species comparisons at corresponding time points."*

3) Some panels and fonts of the illustrations are too small to be readable in printed form. For example, Figs 5c1, c2 and Fig 6b.
Our reply: We have checked and increased the small font size in figures.

Reply to the editor:

Editor: Your manuscript entitled "Conserved Kir channel mechanisms governing intrinsic excitability in human and rodent parvalbumin neurons" has now been seen by 3 referees, whose comments are appended below. You will see from their comments below that while they find your work of considerable interest, some important points are raised. We are interested in the possibility of publishing your study in Communications Biology, but would like to consider your response to these concerns in the form of a revised manuscript before we make a final decision on publication. We therefore invite you to revise and resubmit your manuscript, taking into account the points raised by Reviewer #3. We would suggest adding a proper limitations section (with an appropriate heading) at the end of your discussion and highlighting the points raised by Reviewer #3. Please add here and reiterate that it is difficult to clearly identify PV-positive neurons in human tissue.

Our reply: We respectfully disagree with the reviewer's claim that the Pvalb neuron identification is not sufficiently robust. The study used two independent and widely accepted approaches: immunohistochemical detection of parvalbumin protein and full-transcriptome–based classification obtained through patch-sequencing. Immunolabeling for parvalbumin is a longstanding gold-standard method for identifying Pvalb interneurons, while transcriptomic classification relies on multidimensional gene-expression profiles rather than a single marker, providing a highly specific and internally validated cell-type assignment. In acute human slice experiments, systematic confirmation of every recorded neuron using both methods is technically impractical and would introduce substantial sampling bias. Importantly, neurons classified as Pvalb cells by either approach exhibited the expected electrophysiological phenotype, demonstrating convergence between molecular identity and functional properties. Thus, the identification strategy is methodologically sound, consistent with current standards in human cellular neuroscience, and fully supports the conclusions drawn in the manuscript.

Editor: You may indicate that the cells you recorded from are putative PV-positive neurons.

Our reply: We disagree with the characterization that the recorded cells were merely putative Pvalb neurons. Although not all cells were identified using the identical method—either immunohistochemical detection of parvalbumin protein or full transcriptomic profiling via patch-sequencing—both approaches are well-established and were used in parallel in this study. Specifically, the reviewer questions the identity of the mRNA-based patch-sequenced cells. As noted in the revised limitations section, single–time point patch-sequencing may fail to detect mRNA of genes whose protein is abundantly expressed but whose transcript levels are transiently low due to burst-like transcription dynamics. For this reason, relying on a single marker gene (e.g., PVALB mRNA alone) is not recommended. Instead, following the Allen Institute framework, we based cell-type classification on the full transcriptomic profile—up to ~8,000 detected genes in human Pvalb neurons—and assigned identity using coordinated expression of multiple subtype-specific marker genes. For each human neuron, the identified Pvalb subtype is explicitly shown in the TPM heatmaps presented in the manuscript.

The reviewer also questions the specificity of the mouse Pvalb-Cre;TdTomato line. These mice are widely used and have been validated for parvalbumin specificity by immunohistochemistry, including in our previous studies. Importantly, in the present study we further validated this approach by patch-sequencing ten fluorescent mouse neurons. Nine of ten expressed Pvalb mRNA directly. The one cell without detectable Pvalb transcript expressed multiple additional canonical marker genes characteristic of Pvalb interneurons and was classified accordingly using full transcriptomic profiling. The Allen Institute–based subtype classification is shown for all patch-sequenced human and mouse neurons in the gene expression heatmaps. Taken together, both protein-based and full-transcriptome–based identification methods converge and provide strong molecular evidence that the analyzed cells are bona fide Pvalb interneurons, not merely putative ones.

Editor: Likewise, please add the point raised about ZD7288 here as well (concentrations too high and artifical situation for measuring Rin).

Our reply: Our conclusions do not depend on the absolute physiological state under ZD7288, but on comparative measurements obtained under identical conditions across species. Recording baseline properties without ZD7288 and applying it only before Ba^{2+} would address a different question—the physiological contribution of HCN channels—rather than the intended mechanistic isolation of Kir function (see Szegedi V, et al. HCN channels at the cell soma ensure the rapid electrical reactivity of fast-spiking interneurons in human neocortex. PLoS Biol 21, e3002001, 2023). Thus, applying ZD7288 from the outset was a deliberate strategy to isolate Kir-dependent conductances, reduce confounding influences, and ensure stable and directly comparable recording conditions throughout the experiments.

Importantly, the micromolar concentrations of ZD7288 used here are standard in the literature and effectively block HCN channels without altering Kir function. While off-target effects on other conductances cannot be fully excluded, any such actions are expected to affect human and mouse neurons equally under our identical experimental conditions, and therefore do not change the core finding that Kir-mediated rectification and its proportional similarity are conserved across species.

Reviewers' comments:

Reviewer #3 (Remarks to the Author):

Unfortunately, I do not believe that the concerns raised in my previous review have been adequately addressed.

1) Although the study is of interest, particularly due to the use of human tissue, the results remain relatively limited in scope and impact. In my view, the work would be more suitable for a specialized journal.

Our reply: While we acknowledge the reviewer's perspective, we respectfully disagree that the study is of limited scope or impact. Our work directly addresses a fundamental and conserved mechanism—Kir channel–mediated regulation of intrinsic excitability—in a major interneuron class across humans and rodents, combining electrophysiology, dynamic clamp, patch-sequencing, immunofluorescence, and computational modeling. This multi-modal, cross-species approach provides mechanistic insight that is broadly relevant to understanding cortical circuit function, species differences in excitability, and translational neuroscience, rather than being narrowly descriptive. The use of human tissue allows us to confirm conserved principles in a context that is highly relevant for neurophysiology and potential therapeutic targeting, elevating the study well beyond a specialized audience.

2) I remain concerned that the use of different criteria for identifying parvalbumin-positive cells reduces the rigor of the study. If extended electrophysiological recordings precluded single-cell gene expression profiling, parvalbumin immunostaining could have been applied consistently across all cells. Furthermore, comparing native parvalbumin expression in human neurons with tdTomato fluorescence driven by the parvalbumin promoter in mouse neurons remains suggestive rather than conclusive.

Our reply: We respectfully disagree that the use of complementary identification methods reduces rigor. In human tissue, systematic dual identification of the same neuron by patch-sequencing and post hoc immunostaining is methodologically very challenging due to the prolonged recordings. Instead, neurons were reliably identified using either full-transcriptome patch-sequencing—analyzed against tens of marker genes and classified via the Allen Institute framework—or immunohistochemical detection of Pvalb protein, both widely accepted approaches. In mice, tdTomato expression in Cre-Pvalb animals has been validated against Pvalb protein, and we further confirmed identity by patch-sequencing ten fluorescent cells, nine of which expressed Pvalb mRNA, with the remaining cell validated via additional marker genes. The Allen Institute–based subtype is shown for all patch-sequenced human and mouse neurons in the gene expression heatmaps. Together, these methods provide robust, complementary, and conclusive identification of Pvalb neurons across species.

3) I maintain that measuring input resistance (Figure 1) in the presence of ZD7288 creates a highly artificial and non-physiological condition, as detailed in my original review. While I understand the rationale for using ZD7288 in separate experiments to isolate Kir channel effects during Ba^{2+} application, this does not justify its use in the experiments shown in Figure 1. An alternative approach would have been to record baseline properties without ZD7288 (for Figure 1) and then apply it prior to Ba^{2+} (For Figure 2).

Our reply: Our conclusions do not depend on the absolute physiological state under ZD7288, but on comparative measurements obtained under identical conditions across species. Recording baseline properties without ZD7288 and applying it only before Ba^{2+} would address a different question—the physiological contribution of HCN channels—rather than the intended mechanistic isolation of Kir function (see Szegedi V, et al. HCN channels at the cell soma ensure the rapid electrical reactivity of fast-spiking interneurons

in human neocortex. PLoS Biol 21, e3002001, 2023). Thus, applying ZD7288 from the outset was a deliberate strategy to isolate Kir-dependent conductances, reduce confounding influences, and ensure stable and directly comparable recording conditions throughout the experiments.

4) The section on mRNA expression of Kir channel subunits remains weak. As the authors acknowledge, mRNA levels may not reflect protein expression, and the conclusion that human and mouse parvalbumin neurons express multiple Kir channel types is modest. The functional significance of this diversity is not discussed.

Our reply: We added in discussion the following senstenses: "Mouse Pvalb neurons rely on multiple Kir subtypes for flexible inhibition in fast circuits. Human Pvalb neurons achieve the required level of inward rectification and gain control with fewer Kir subtypes indicating that additional molecular diversity offers limited incremental benefit. The reduced Kir subtype diversity in human Pvalb neurons may reflect functional optimization rather than limitation. Given the larger size, higher input resistance, and distinct scaling properties of human neurons, proportional Kir-mediated gain control can be achieved with fewer molecular components with less molecular and pharmacological diversity. Such simplification may represent a neuroeconomic strategy that minimizes metabolic cost while preserving function with energy-efficient circuit design. This aligns with broader principles of large brain energy optimization."

5) The computational modeling and dynamic clamp experiments were conducted with HCN channels absent or blocked, again representing a non-physiological condition. Although consistency across experiments is desirable, this should have been achieved by performing the measurements in Figure 1 and the modeling/dynamic clamp experiments in Figure 7 under control conditions (with intact HCN conductance) rather than under HCN blockade.

Our reply: We respectfully disagree that the use of HCN channel blockade undermines the validity of our computational and dynamic-clamp results. The primary aim of these experiments was to isolate Kir-mediated effects on input resistance and synaptic integration; leaving HCN channels active would have introduced variable, voltage-dependent currents that could obscure the direct contribution of Kir channels. The suggestion to record baseline properties first under intact HCN conductance addresses a different question—the contribution of HCN currents—rather than the mechanistic question we aimed to address. Importantly, micromolar ZD7288 concentrations, while standard in slice experiments, have not altered the proportional Kir-dependent rectification observed in human versus mouse Pvalb neurons, demonstrating that our central conclusions are directly attributable to Kir function, independent of HCN activity.
